# Turn-on Coumarin Precursor: From Hydrazine Sensor to Covalent Inhibition and Fluorescence Detection of Rabbit Muscle Aldolase

**DOI:** 10.3390/molecules29102175

**Published:** 2024-05-07

**Authors:** Sara Amer, Uri Miles, Michael Firer, Flavio Grynszpan

**Affiliations:** 1 Department of Chemical Sciences, Ariel University, 65 Ramat HaGolan Street, Ariel 4077625, Israel; 2 Department of Chemical Engineering and Biotechnology, Ariel University, Ariel 4077625, Israel; firer@ariel.ac.il; 3 Adelson School of Medicine, Ariel University, Ariel 4077625, Israel

**Keywords:** hydrazine sensor, turn-on, fluorescent detection, coumarin, aldolase inhibitor, theranostics

## Abstract

Hydrazine, a highly toxic compound, demands sensitive and selective detection methods. Building upon our previous studies with pre-coumarin OFF–ON sensors for fluoride anions, we extended our strategy to hydrazine sensing by adapting phenol protecting groups (propionate, levulinate, and γ-bromobutanoate) to our pre-coumarin scaffold. These probes reacted with hydrazine, yielding a fluorescent signal with low micromolar limits of detection. Mechanistic studies revealed that hydrazine deprotection may be outperformed by a retro-Knoevenagel reaction, where hydrazine acts as a nucleophile and a base yielding a fluorescent diimide compound (6,6′-((1*E*,1′*E*)-hydrazine-1,2diylidenebis(methaneylylidene))bis(3(diethylamino)phenol, **7**). Additionally, our pre-coumarins unexpectedly reacted with primary amines, generating a fluorescent signal corresponding to phenol deprotection followed by cyclization and coumarin formation. The potential of compound **3** as a theranostic Turn-On coumarin precursor was also explored. We propose that its reaction with ALDOA produced a γ-lactam, blocking the catalytic nucleophilic amine in the enzyme’s binding site. The cleavage of the ester group in compound **3** induced the formation of fluorescent coumarin **4**. This fluorescent signal was proportional to ALDOA concentration, demonstrating the potential of compound **3** for future theranostic studies in vivo.

## 1. Introduction

Hydrazine is a very toxic compound with serious health implications for humans. It causes irritation to the eyes, respiratory track and the skin. It is rapidly absorbed through the skin and affects the nervous system resulting in an upset stomach, body shaking, lethargy and neuritis [1,2,3,4,5]. It is also a suspected carcinogen [2]. Most commonly, exposure to hydrazine comes from intake of contaminated water or from eating tainted fish [6,7]. Hydrazine is commonly used in agricultural pesticides, as an intermediate reagent in the pharmaceutical industry, as a photography reagent, and as rocket fuel [8]. The identification and quantification of hydrazine in various environments remains a relevant task. Over the years, several chemical sensors for hydrazine have been developed [9,10,11]. Turn-on fluorescent detection of hydrazine is probably the most attractive method and some examples in both environmental and biological systems have been reported [12,13,14,15,16,17,18].

Recently, we described the design, synthesis, and implementation of a fluoride chemosensor based on the use of a non-fluorescent pre-coumarin derivative. The designed probe undergoes selective fluoride-triggered coumarin formation accompanied by a turn-on fluorescence signal. The use of this probe is convenient, displays excellent selectivity and its sensitivity was the lowest reported for this sensor class [19]. While studying the mechanism of the pre-coumarin intramolecular cyclization we encountered the need for analogs bearing different trigger response units. Thus, based on the literature precedents of N_2_H_4_ sensitive protecting groups, we designed hydrazine sensing probes **1**, **2**, and **3** in which the triisopropyl siloxy group of the fluoride sensor was replaced by a propionate, a levulinate and a γ-bromobutanoate, respectively. Compound **3** allowed us to elucidate the in situ alkene isomerization of the major *E*-isomer precursor to the reactive *Z*-isomer, which occurs during coumarin formation [16]. Compounds **1**, **2**, and **3** were tested for the detection of hydrazine with LOD values in the range of previously reported chemical sensors bearing parallel triggering moieties [15,16,17,18]. In some studies, the sensors’ response to primary amines was unsuccessful at low concentrations [20], while in others there was no response even upon the addition of 50 or 80 equiv. of the amine [21,22]. In our hands, the reaction with a primary amine (ethylamine) was observed with the addition of only a 30 equivalent of this nucleophile.

After noting the spectroscopic response of compounds **1**, **2**, and **3** in the presence of a primary amine, we performed a short preliminary study and exposed compound **3**, which appeared to afford the best results in terms of fluorescence response, to a nucleophilic amine at physiological pH. Fructose-1,6-bisphosphate aldolase (aldolase A or ALDOA), a Class I aldolase, is involved in the glycolytic pathway and reversibly catalyzes the cleavage of fructose 1,6-bisphosphate into the triose phosphates d-glyceraldehyde phosphate and dihydroxyacetone phosphate [19]. Cancer cells take up glucose and supply their energy requirements through anaerobic glycolysis; in these cells, ALDOA is overexpressed [23]. Indeed, certain ALDOA inhibitors have demonstrated antitumor activity [24,25]. Another attractive application of fluorescent probes is their utility as theranostic agents [26,27]. The “theranostic” concept has been defined as the application of a compound that combines therapeutic and diagnostic capabilities. A theranostic agent delivers a pharmaceutic and a diagnostic dose simultaneously in one molecular package. This methodology is expected to greatly impact future personalized medicine approaches [28]. In this work, we introduce compound **3** as a potential ALDOA theranostic turn-on coumarin precursor. We propose that the non-fluorescent precursor **3**, bearing a phenol functionality protected by a γ-bromoester group, selectively reacts with a catalytic nucleophilic amine in ALDOA. The S_N_2 reaction results in a secondary amine which undergoes an intramolecular cyclization reaction to produce a γ-lactam that covalently inhibits the enzyme. The cleavage of the ester group releases the phenol that induces the formation of the fluorescent coumarin **4**, which can be easily detected while it diffuses away from the binding site. In principle, this theranostic process could enable the therapeutic inhibition of ALDOA in cancer cells and its concomitant quantitative diagnostic detection by tracking the fluorescence response. In vivo tests to confirm this hypothesis are beyond the scope of this study and will be performed in due course.

## 2. Results and Discussion

**Figure 1:**
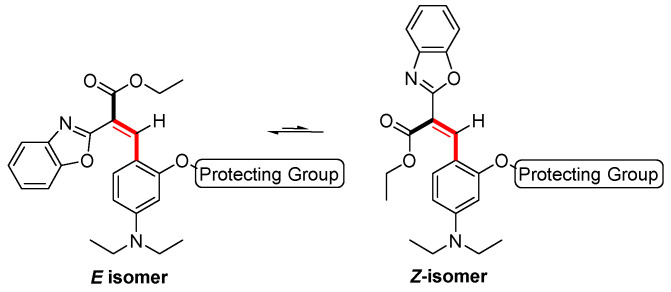
Representative *E* to *Z* isomers as seen in all pre-coumarins.

During the course of the multi-step organic syntheses, it is common to use protecting groups which temporarily block a reactive site on a synthetic intermediate. The deprotection step is then usually carried out efficiently under highly specific conditions [29]. This “protection–deprotection” approach inspired the design of a variety of fluorescent sensors where the target molecule presents different photophysical properties before and after the molecular recognition event [30,31,32,33]. Based on this general idea, we reported a fluoride chemosensor (with the lowest reported limits of detection for this sensor class) using a non-fluorescent phenol protected pre-coumarin. This compound undergoes selective fluoride deprotection of a triisopropyl silyl ether group and the formation of a coumarin turning ON a fluorescence signal [19]. The mentioned pre-coumarin sensor exists as a mixture of alkene isomers (2:1 in favor of the *E* isomer, Figure 1). Of note, only the minor *Z*-isomer can undergo cyclization to the fluorescent coumarin. In order to provide experimental evidence for the in situ *E* to *Z* isomerization, we designed three pre-coumarins protected by reported chemoselective hydrazine recognizing groups [34,35,36].

**Figure 2:**
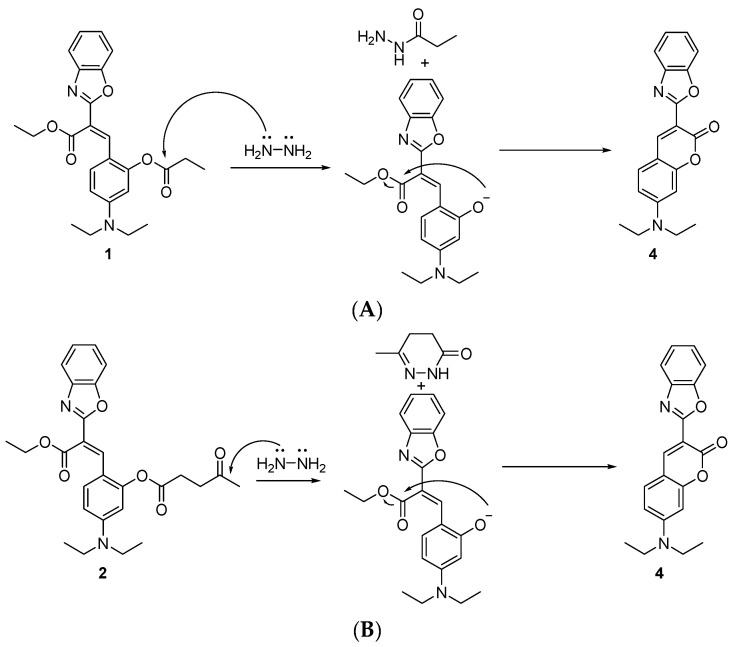

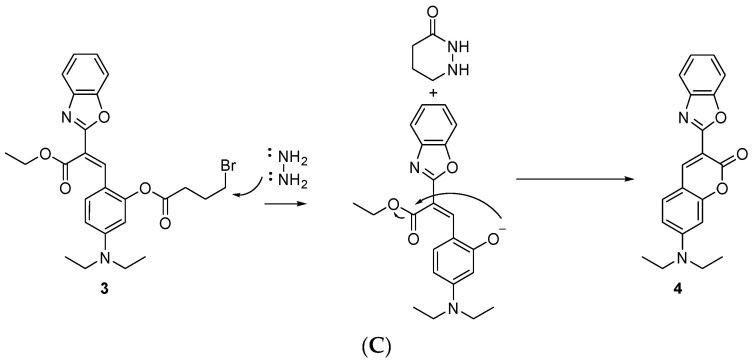
(**A**–**C**). Expected reactions of hydrazine with pre-coumarins **1**, **2**, and **3**.

Thus, pre-coumarin propionate (**1**), levulinate (**2**) and γ-bromobutanoate (**3**) derivatives were prepared. Following previous reports, it was expected that after the cleavage of the protecting group in the non-fluorescent precursors an intramolecular cyclization reaction would generate a closed and fluorescent coumarin derivative (Figure 2A–C).

**Figure 3:**
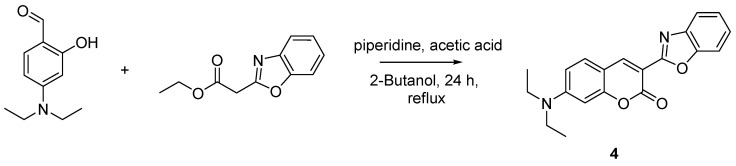
Synthetic scheme followed to prepare the closed coumarin derivative (compound **4**).

An independently prepared sample of this coumarin derivative (**4**), as previously reported, was synthesized for comparison purposes following a Knoevenagel condensation (Figure 3). This “push–pull” coumarin includes a donor group (diethylamine) and an acceptor group (benzoxazole) strategically located on the π-conjugated backbone, which enables intramolecular charge transfer (ICT) [37]. The calculated quantum yield (*ϕ*) for compound **4**, a very suitable reporter unit, is 0.78 (2) and its extinction coefficient (ε) is 50,276 (315) M^−1^ cm^−1^ in acetonitrile solutions [38].

The Knoevenagel reaction of ethyl 2-(benzo[d]oxazol-2-yl)acetate and the phenol protected 5-(diethylamino)-2-formylphenyl derivative affords circa. 2:1 mixtures of *E* and *Z* pre-coumarins **1**, **2**, and **3**. As we reported previously, the *E* to *Z* isomerization process facilitates the ring closing of the *Z* deprotected phenol to the fluorescent coumarin **4**, consuming the whole mixture of starting materials [19].

The abilities of compounds **1**, **2** and **3** to detect N_2_H_4_ were evaluated by measuring their corresponding absorbance and emission spectra at a 10 μM concentration of the pre-coumarin dissolved in water–acetonitrile (*v*/*v* = 1:9) in the presence of increasing concentrations of N_2_H_4_ (0–400.0 μM).

The emission intensity at 494 nm of **1**, **2** and **3** gradually increased up to 27, 22 and 29 -fold, respectively, upon the addition of N_2_H_4_ portions while with the absorbances at 400 nm decreased by about 2-fold in all cases and were accompanied by a blue shift of 15, 0 and 42 nm, respectively. The hydrazine limits of detection (LOD) of 1.8 (3), 2.7 (2), and 0.84 (1) μM were calculated for **1**, **2** and **3**.

Our three probes did not excel as sensors for hydrazine and exhibit LOD values comparable to other previously reported sensors of this class. It could be presumed the rate limiting steps of our sensors, namely their reaction with hydrazine, are similar to those reported in the literature [33,39].

To evaluate the selective response of compounds **1**, **2** and **3** towards N_2_H_4_, we studied the effect of typical anions (Cl^−^, Br^−^, I^−^, F^−^, NO_3_^−^ and ClO_4_^−^), cations (Na^+^, K^+^, Mg^2+^, Ni^2+^, Co^2+^ and Cu^2+^), and typical biological and organic nucleophilic analytes (Alanine, Cysteine, Lysine, ethylamine, 2-aminothiophenol and thiourea) on the absorbance and fluorescence behavior of our pre-coumarin compounds in water–acetonitrile solutions (*v*/*v* = 1:9).

Solutions of **1**, **2** or **3** (10 μM) were prepared, and the analytes listed above were added at a concentration of 300 μM. Of note, when hydrazine or ethylamine were added to compounds **1**, **2** and **3**, the fluorescence intensity increased significantly. The addition of 300 μM lysine, or alanine, only resulted in a slight fluorescence increase (See Supplementary Materials). The reaction with ethylamine, a primary amine, was readily observed with the addition of only 30 equiv. of the analyte. These observations depart from the literature results where additions of 50 [21] or 80 [40] equiv. of primary amine analytes did not produce any observable response in the studied systems.

The proposed reaction mechanisms reported for phenol release from these protecting groups include the cleavage of acetoxy group and hydrazide formation from **1** (Figure 2A), nucleophilic addition–elimination on the keto-ester followed by the cyclization of the enamine form to the corresponding pyrrolone from **2** (Figure 2B) and nucleophilic substitution–elimination on the γ-bromoester followed by cyclization to pyrrolidone (a γ-lactam) from **3** (Figure 2C) [9,41]. In all three cases, the free phenol in the *Z* isomer is expected to rapidly cyclize to the corresponding coumarin **4**. As predicted, the fluorescence spectra of **1**, **2**, and **3** after hydrazine addition (30 equiv.) overlapped with that of **4**. To further analyze these mechanisms, the fluorescent products were submitted to HRMS, aiming to achieve the mass confirmation for compound **4** (MW = 334.3750 gr/mol). Interestingly, molecular peaks at *m*/*z* = 383.2447 were found in all three samples. In addition, the reaction of **2** with increasing amounts of hydrazine was followed by ^1^H NMR (See Supplementary Materials for spectra). A comparison of these spectra to that of an independent sample of **4** clearly indicated that the expected coumarin was not the product of these reactions.

**Figure 4:**
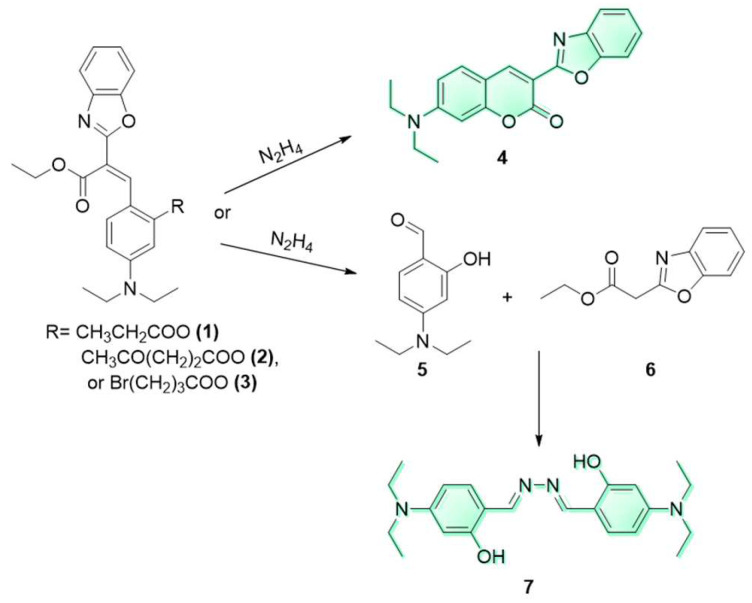
Proposed pathways and products upon addition of excess N_2_H_4_ to compounds **1**, **2** or **3**.

The crude products of each reaction were subjected to column chromatography which returned two isolated non-fluorescent materials and one fluorescent compound. The full characterization of these products indicated that the reaction with hydrazine induced the nucleophilic/basic decomposition of all three pre-coumarins to the non-fluorescent starting materials **5** and **6** by a retro Knoevenagel reaction [42], and the formal subsequent reaction of hydrazine with two equiv. of the aldehyde (for a suggested mechanism, see the Supplementary Materials). The bis-Schiff base **7** is fluorescent and its MW = 383.30 gr/mol. Compound **7** has been previously characterized by others [43], and our spectroscopic data for it is in full agreement with the reported data (Figure 4).

To further corroborate these results, we prepared pre-coumarins **8**, **9** and **10** and coumarin **11**. Not surprisingly, upon reaction with hydrazine, these three pre-coumarins only produced small amounts of the fluorescent **11**, whereas the fluorescent diimine **7** was again the main product. The retro-Knoevenagel reaction in **8**, **9** and **10** is expected to be slower than that for compounds **1**, **2**, and **3**, explaining the formation of the small amount of **11** following the deprotection and cyclization reaction path.


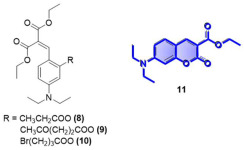


**Figure 5:**
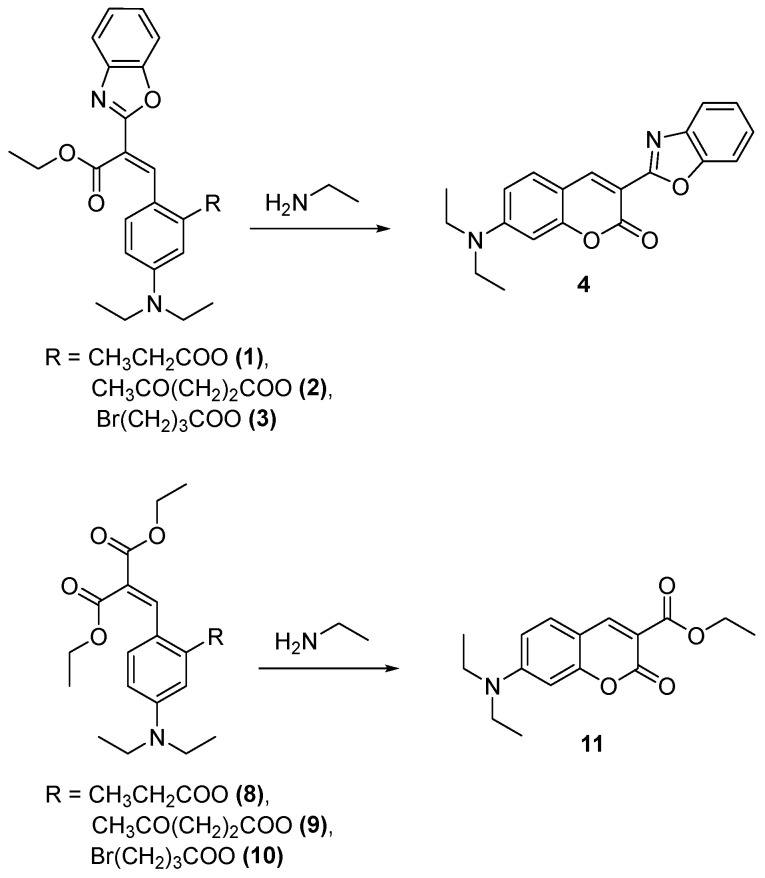
Reactions of compounds **1**, **2**, **3**, **8**, **9** and **10** with excess ethylamine.

Pre-coumarins **1**, **2**, and **3** also react with primary amines (*vide supra*). ^1^H NMR titration was carried out to reveal the chemical nature of the observed fluorescence response. A sample of **3** (10 mg, 0.021 mmol) in CDCl_3_ (0.6 mL) was prepared and its 1D ^1^H NMR spectrum was acquired. Two portions of ethylamine (1.0 equiv. and 30.0 equiv.) were added to this sample. The ^1^H NMR spectra clearly shows that only chemical shifts ascribed to compound **4** begin to appear. To confirm this result, LC-MS tests were performed on all six pre-coumarins. Thus, 1.0 mM samples of compounds **1**, **2**, **3**, **8**, **9** and **10** in water–acetonitrile solutions (*v*/*v* = 1:9) were prepared. To each sample, 30.0 equiv. of ethylamine were added, and the reaction followed by UV-Vis and fluorescence spectra (See Supplementary Materials). The LC-MS results showed that the reactions with ethylamine resulted only in the formation of the corresponding fluorescent coumarin **4** with no evidence for the formation of diimine **7** under these reaction conditions (Figure 5).

**Figure 6:**
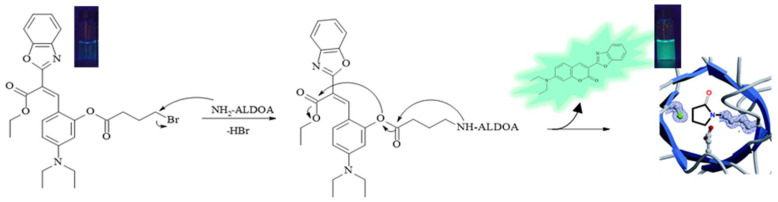
Proposed inhibition mechanism of ALDOA and release of fluorescent coumarin **4**.

Nucleophilic primary amines can be found in the binding sites of Class I aldolase enzymes, which are common in animals and higher plants. In their catalytic clefts, these enzymes present a lysine residue bearing an ε-amino group with a disturbed p*K*_a_ as low as 7.7 [44]. These enzymes work via Schiff-base formation with their carbonyl substrate. Fructose-1,6-bisphosphate aldolase, a Class I aldolase, is involved in the glycolytic pathway and reversibly catalyzes the cleavage of fructose 1,6-bisphosphate into the triose phosphates δ-glyceraldehyde phosphate and dihydroxyacetone phosphate [22]. These Class I aldolases can be classified into three isoenzyme forms: ALDOA, ALDOB, and ALDOC. Among the three Class I aldolase isoenzymes, ALDOA from rabbit muscle, has been the most widely studied [19]. As mentioned previously, the overexpression of ALDOA has been observed in various cancers including lung, renal cell and hepatocellular carcinoma [23] and may represent a valid therapeutic target. Based on the reaction between our pre-coumarins and ethylamine, we asked whether compound **3**, which produced the quickest fluorescence response, could potentially react with ALDOA’s nucleophilic primary amine acting as a simultaneous covalent inhibitor and sensor (Figure 6). This non-fluorescent precursor (compound **3**) has a phenol functionality protected by a γ-bromobutyl ester group ready to react with the catalytic nucleophilic amine in ALDOA. This S_N_2 reaction results in a secondary amine which undergoes an intramolecular cyclization reaction to produce a γ-lactam covalently inhibiting the enzyme. The cleavage of the ester group induces the formation of the fluorescent coumarin **4** which diffuses away from the binding site. In principle, this theranostic process could enable the therapeutic inhibition of ALDOA in cancer cells and its concomitant quantitative diagnostic detection by tracking the fluorescence response.

**Figure 7:**
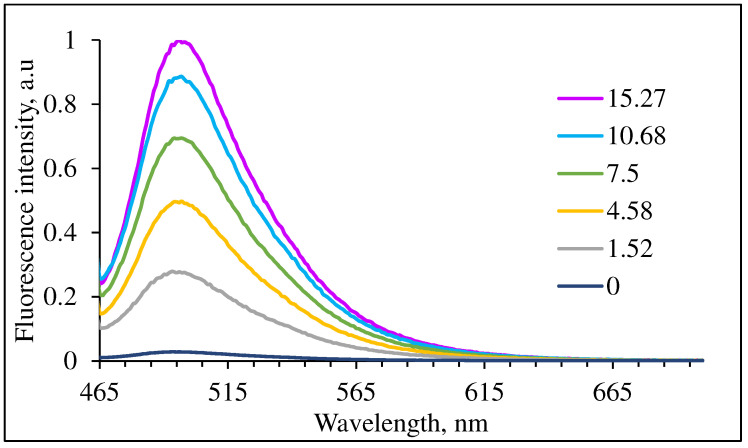
Normalized fluorescence of 5.0 μM **3** in 1% MeCN–99% PBS buffer pH 7.4 upon addition of 0–15.27 nM ALDOA (λ_ex_ = 455 nm).

The reaction of **3** (5.0 μM) in the presence of six ALDOA samples with increasing concentrations (0–15.27 nM) was studied using 1% MeCN: 99% PBS buffer at physiological pH. The fluorescence intensity increases with the addition of ALDOA as illustrated in Figure 7. The LOD of ALDOA with compound **3** was calculated as 0.063 (6) nM.

**Figure 8:**
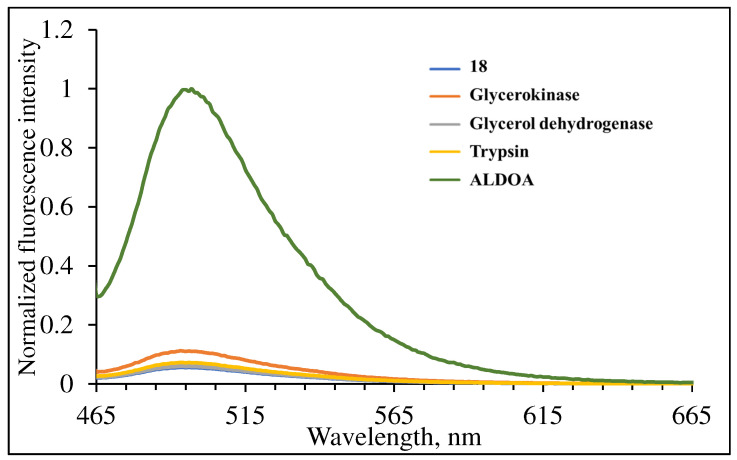
Normalized fluorescence of 5.0 μM **3** in 1% MeCN: 99% PBS buffer pH 7.4 upon addition of 4.58 nM enzyme. (λ_ex_ = 455 nm).

To challenge the selectivity of **3** towards ALDOA, we exposed a 5.0 μM sample of **3** to glycerol kinase, trypsin and glycerol dehydrogenase. Glycerol kinase is an enzyme that catalyzes the transfer of a phosphate from ATP to glycerol [45]. Trypsin is a serine protease that cleaves the peptide bond in the carboxylic side of lysine and arginine residues [46]. Glycerol dehydrogenase is an oxidoreductase enzyme that uses NAD^+^ to catalyze the oxidation of glycerol to dihydroxyacetone [47]. The studies were conducted in 1% MeCN: 99% PBS buffer, with a of pH 7.4 upon the addition of 4.58 nM of each enzyme. The response of **3** towards these three enzymes is negligible indicating the selective activity of **3** for ALDOA (see Figure 8).

## 3. Materials and Methods

NMR spectra (^1^H and ^13^C) were recorded using a Bruker Avance-III 400 MHz spectrometer, equipped with a 5 mm BBFO SmartProbe; the spectra were processed with MestReNova v6.0.2-5457 (Mestrelab Research, Santiago de Compostela, Spain). Liquid chromatography mass spectrometry (LC-MS) was performed using an Aglient Technologies 6120 Quadrupole LC/MS; mobile phase A was 0.1% formic acid in water and mobile phase B was acetonitrile. The flow rate was 0.03 mL/min and the run time was set to 23 min. The products were separated and analyzed using standard methods of chromatography (Thin Layered Chromatography, Column Chromatography, and medium-pressure chromatography (Flash Chromatography)) using Silica Gel (60 mesh) as the stationary phase.

The samples were placed in a 10 mm pathlength absorbance quartz cuvette. Fluorescence spectra were recorded on a Varian Cary Eclipse Fluorescence Spectrophotometer, using a 2.5 nm excitation slit width, a 5 nm emission slit width, and a 120 nm/min scan rate. No corrections were applied to the measured emission spectra. The UV-vis absorbance spectra were measured with a Varian Cary 100 Bio UV-vis spectrophotometer, using a 10 mm pathlength quartz cuvette and scan rate of 600 nm/min.

Quantum yields were calculated by the single point relative quantum yield method, using Equation (1) [48].

(1) $$\phi_{F(x)}={\left(\frac{n_{x}}{n_{s}}\right)}^{2}(\frac{A_{s}}{A_{x}})(\frac{I_{f(x)}}{I_{f(s)}})\phi_{F(s)}$$

where *ϕ_F_* is the fluorescence quantum yield, *A* is the absorbance, *I_f_* is the integration of the fluorescence band, and *n* is the solvent refractive index. The subscripts *s* and *x* refer to the standard and unknown samples, respectively [24]. In this work, the fluorescence quantum yield standards were solutions of 10 or 20 μM of 1,4-bis(5-phenyloxazol-2-yl)benzene (POPOP) in cyclohexane. For these solutions, *ϕ_F_* = 0.97 [49].

The limit of detection (LOD) was determined from a graph which was plotted with integrated emission (vs. wavenumber) on the *Y*-axis and concentration of the analyte (in M) on the *X*-axis, from which an equation for the resulting straight line was determined. The limit of detection (LOD) was defined according to Equation (2):

LOD = 3σ/K(2)

where σ is the standard deviation of the blank measurement, and K is the slope between integrated emission versus analyte concentration.

### Synthetic Procedures

**Ethyl 2-(benzo[d]oxazol-2-yl)acetate. Compound 6:** 2-Aminophenol (1 g, 9 mmol, 1equiv.) was placed to in a 100 mL flask. Diethylmalonate (4.32 g, 15 mmol, 15 equiv.) was added followed by *p*-toluene sulfuric acid (1.55 g, 9 mmol, 1 equiv.). Nitrogen was flushed for 15 min. The reaction mixture was refluxed overnight under nitrogen atmosphere. Completion of reaction was monitored using TLC. The product was purified using column chromatography starting with hexane 80%:EtOAc 20%, ethyl 2-(benzo[d]oxazol-2-yl)acetate was eluted with hexane 50%:EtOAc 50% to give a yellow oil (674 mg, 36% yield). ^1^H NMR (400 MHz, CDCl_3_): δ 1.22 (dd, 3H, *J* = 8.9, 5.4 Hz), 3.97 (m, 2H), 4.19 (q, 2H, *J* = 7.1 Hz), 7.25 (m, 2H), 7.45 (m, 1H), 7.65 (m, 1H) ppm. ^13^C NMR (100.61 MHz, CDCl_3_): δ 14.15, 35.20, 61.77, 110.29, 119.62, 124.31, 125.03, 141.26, 150.96, 159.54, 166.89 ppm. LC-MS, M+H^+^, *m*/*z* Calc. 205.21, Found 206.10.


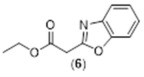


**3-(Benzo[d]oxazol-2-yl)-7-(diethylamino)-2H-chromen-2-one. Compound 4**: Diethylamino salicylaldehyde (270 mg, 1.3 mmol, 1.0 equiv.) was dissolved in 10 mL of 2-Butanol in a 25 mL flask., ethyl 2-(benzo[d]oxazol-2-yl)acetate (230 mg, 2 mmol, 1.5 equiv.) was added to this solution, followed by piperidine (47 mg, 0.5 mmol, 0.4 equiv.). Acetic acid (33 mg, 0.5 mmol, 0.4 equiv.) was then added to the reaction mixture which was refluxed for 24 h. The completion of the reaction was monitored using TLC. The precipitate was filtered and washed three times with 2-Butanol, and dried under reduced pressure to give compound **4** as a pale orange solid (242 mg, 52% yield). ^1^H NMR (400 MHz, CDCl_3_): δ 1.25 (m, 6H), 3.44 (m, 4H), 6.53 (d, 1H, *J* = 2.3 Hz), 6.64 (dd, 1H, *J* = 8.9, 2.5 Hz), 7.26 (s, 1H), 7.33 (m, 1H), 7.41 (m, 1H), 7.58 (m, 1H), 7.79 (m, 1H), 8.6 (s, 1H) ppm. ^13^C NMR (100.61 MHz, CDCl_3_): δ 12.59, 45.27, 97.14, 106.66, 108.35, 109.87, 110.40, 120.23, 124.64, 124.87, 130.69, 142.22, 145.04, 150.53, 152.09, 158.04, 158.23, 160.03 ppm. LC-MS, M+H^+^, *m*/*z* Calc. 334.47, found 335.10.


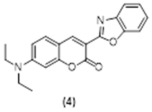


**5-(Diethylamino)-2-formylphenyl propionate. Compound 5:** Diethylaminesalicaldehyde (1g, 5.0 mmol, 1equiv.) was added to a 100 mL flask which was flushed with nitrogen for 15 min. Acetonitrile (dry, 20 mL) was introduced and then propionic acid (382 mg, 5.0 mmol, 1.0 equiv.) was added followed by DMAP (630 mg, 1.0 mmol, 0.2 equiv.) and EDC (1.2 g, 7.5 mol, 1.5 equiv.). The reaction mixture was stirred for 24 h at room temperature. The completion of reaction was monitored using TLC. The product was purified using column chromatography starting with EtOAc 10%:Hexane 90%, and the desired product was eluted with EtOAc 30%:Hexane 70% to give compound **5** as a yellow oil (565 mg, 44% yield). ^1^H NMR (400 MHz, CDCl_3_): δ 1.08 (m, 6H), 1.19 (dd, 3H, *J* = 8.5, 6.6 Hz), 2.56 (m, 2H), 3.29 (q, 4H, *J* = 7.1 Hz), 6.18 (d, 1H, *J* = 2.5 Hz), 6.44 (dd, 1H, *J* = 8.9, 2.5 Hz), 7.54 (m, 1H), 9.65 (s, 1H) ppm. ^13^C NMR (100.61 MHz, CDCl_3_): δ 8.75, 12.17, 27.38, 44.53, 104.23, 108.21, 115.96, 133.41, 152.91, 153.33, 172.54, 186.26 ppm.


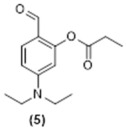


**Ethyl-2-(benzo[d]oxazol-2-yl)-3-(4-(diethylamino)-2-(propionyloxy)phenyl)acrylate, Compound 1 (*E*+*Z*):** 5-(Diethylamino)-2-formylphenyl propionate (**5**, 820 mg, 3.3 mmol, 1.0 equiv.) was dissolved in dichloromethane (DCM anh., 20 mL) in a 50 mL round-bottomed flask. Ethyl 2-(benzo[d]oxazol-2-yl)acetate (**6**, 1.35 g, 6.58 mmol, 2.0 equiv.) was added to this solution together with piperidine (112 mg, 1.32 mmol, 0.4 equiv.), and acetic acid (79 mg, 1.32 mmol, 0.4 equiv.). The reaction mixture was refluxed overnight. The mixture was cooled to room temperature and the solvent was removed under reduced pressure. The crude product was purified using preparative HPLC using an acetonitrile 70%:water 30% solvent mixture. Compound **1**, as a mixture of *E* and *Z* isomers, was obtained as a yellow oil (676 mg, 47% yield, ~2:1 *E*: *Z* mixture).


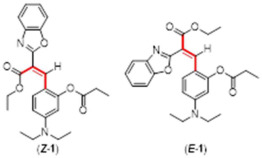


***E*-isomer:** ^1^H NMR (400 MHz, CD_3_CN): δ = 8.05 (s, 1 H), 7.79 (m, 1 H), 7.63 (m, 1 H), 7.45 (m, 1 H), 7.44 (m, 1 H), 6.62 (d, 1 H, *J* = 9.1 Hz), 6.38 (d, 1 H, *J* = 2.7 Hz), 6.26 (dd, 1 H, *J* = 9.1, 2.7 Hz), 4.23 (q, 2 H, *J* = 7.1 Hz), 3.30 (q, 2 H, *J* = 7.1 Hz), 2.63 (m, 3 H), 1.25 (t, 3 H, *J* = 5.4 Hz), 1.22 (t, 3 H, *J* = 7.1 Hz), 1.06 (t, 6 H, *J* = 7.1 Hz) ppm. ^13^C NMR (100.61 MHz, CD_3_CN): δ = 172.56, 165.34, 159.95, 153.77, 151.06, 140.72, 125.62, 124.18, 119.85, 113.59, 110.57, 109.44, 109.09, 60.88, 43.95, 26.91, 13.48, 11.74, 8.51 ppm.

***Z*-isomer:** ^1^H NMR (400 MHz, CD_3_CN): δ = 7.69 (s, 1 H), 7.66 (m, 1 H), 7.59 (m, 1 H), 7.41 (m, 1 H), 7.37 (m, 1 H), 7.36 (m, 1 H), 6.64 (dd, 1 H, *J* = 9.1, 2.7 Hz), 6.45 (d, 1 H, *J* = 2.7 Hz), 4.41 (q, 2 H, *J* = 7.1 Hz), 3.42 (q, 2 H, *J* = 7.1 Hz), 2.63 (m, 3 H), 1.32 (t, 3 H, *J* = 5.4 Hz), 1.25 (t, 3 H, *J* = 7.1 Hz), 1.15 (t, 6 H, *J* = 7.1 Hz) ppm ^13^C NMR (100.61 MHz, CD_3_CN): δ = 172.56, 166.70, 160.94, 150.94, 150.44, 130.87, 129.80, 129.60, 129.16, 124.19, 119.39, 117.26, 110.81, 104.64, 61.55, 43.94, 26.91, 13.52, 13.48, 11.64 ppm.

**5-(Diethylamino)-2-formylphenyl 4-oxopentanoate:** Diethylaminesalicaldehyde (390 mg, 2.0 mmol, 1.0 equiv.) was dissolved in 200 mL of acetonitrile (dry) in a 250 mL round bottom flask which was flushed with nitrogen for 15 min. Then, levulinic acid (580 mg, 5.0 mmol, 2.5 equiv.) was added followed by DMAP (24 mg, 0.2 mmol, 0.2 equiv.). EDC (770 mg, 4 mmol, 2.0 equiv.) was added to the reaction mixture which was stirred for 24 h at room temperature. The completion of the reaction was monitored using TLC. The product was purified using column chromatography starting with EtOAc 20%:Hexane 80%, and the desired product was eluted with EtOAc 70%:Hexane 30% yielding a 546 mg of a yellow oil (93% yield). ^1^H NMR (400 MHz, CDCl_3_): δ 1.19 (m, 6H), 2.18 (m, 3H), 2.59 (m, 2H), 2.72 (m, 2H), 3.38 (q, 4H, *J* = 7.1 Hz), 6.25 (d, 1H, *J* = 2.5 Hz), 6.51 (dd, 1H, *J* = 8.9, 2.5 Hz), 7.60 (m, 1H), 9.68 (s, 1H) ppm. ^13^C NMR (100.61 MHz, CDCl3): δ 12.49, 28.01, 29.89, 37.89, 44.86, 104.61, 108.34, 116.15, 133.73, 153.69, 170.70, 178.12, 187.10, 206.84 ppm.


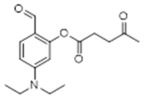


**2-(2-(Benzo[d]oxazol-2-yl)-3-ethoxy-3-oxoprop-1-en-1-yl)-5-(diethylamino)phenyl 4-oxopentanoate, Compound 2:** 5-(Diethylamino)-2-formylphenyl 4-oxopentanoate (530 mg, 1.82 mmol, 1.0 equiv.) was dissolved in dichloromethane (DCM anh., 10 mL) in a 50 mL round bottom flask. Ethyl 2-(benzo[d]oxazol-2-yl)acetate (**6**, (747 mg, 3.63 mmol, 2.0 equiv.), piperidine (62 mg, 0.72 mmol, 0.4 equiv.), and acetic acid (44 mg, 0.72 mmol, 0.4 equiv.) were then added. The reaction mixture was refluxed overnight. After cooling to room temperature and the removal of the solvent under reduced pressure, the product was purified using preparative HPLC using an acetonitrile 70%:water 30% solvent mixture. Compound **2**, as a mixture of *E* and *Z* isomers, was obtained as a yellow oil (383 mg, 44% yield, ~2:1 *E*: *Z* mixture).


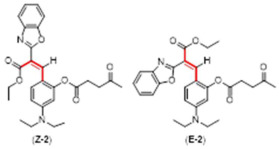


***E*-isomer:** ^1^H NMR (400 MHz, CD_3_CN): δ = 8.06 (s, 1 H), 7.66 (m, 1 H), 7.62 (m, 1 H), 7.36 (m, 1 H), 7.35 (m, 1 H), 6.59 (m, 1 H), 6.35 (d, 1 H, *J* = 2.7 Hz), 6.26 (dd, 1 H, *J* = 10.5, 5.2 Hz), 4.23 (q, 2 H, *J* = 7.1 Hz), 3.29 (q, 2 H, *J* = 7.1 Hz), 2.88 (m, 2 H), 2.81 (m, 2 H), 2.13 (t, 3 H, *J* = 5.4 Hz), 1.24 (t, 3 H, *J* = 7.1 Hz), 1.06 (t, 6 H, *J* = 7.1 Hz) ppm. ^13^C NMR (100.61 MHz, CD_3_CN): δ = 207.53, 172.58, 166.26, 161.11, 153.72, 152.05, 142.06, 131.23, 130.73, 125.53, 120.52, 115.22, 111.45, 110.16, 105.59, 62.21, 45.20, 38.56, 29.75, 28.78, 14.53, 12.67 ppm.

***Z*-isomer:** ^1^H NMR (400 MHz, CD_3_CN): δ = 7.76 (m, 1 H), 7.71 (s, 1 H), 7.62 (m, 1 H), 7.45 (m, 1 H), 7.44 (m, 1 H), 7.39 (m, 1 H), 6.65 (d, 1 H, *J* = 2.7 Hz), 6.43 (d, 1 H, *J* = 2.7 Hz), 4.40 (q, 2 H, *J* = 7.1 Hz), 3.40 (q, 2 H, *J* = 7.1 Hz), 2.88 (m, 2 H), 2.81 (m, 2 H), 2.13 (t, 3 H, *J* = 5.4 Hz), 1.32 (t, 3 H, *J* = 7.1 Hz), 1.15 (t, 6 H, *J* = 7.1 Hz) ppm ^13^C NMR (100.61 MHz, CD_3_CN): δ = 207.53, 172.58, 167.72, 162.71, 152.87, 151.45, 132.33, 130.53, 126.82, 125.24, 120.96, 118.34, 111.45, 110.40, 105.75, 62.72, 45.19, 38.56, 29.75, 28.78, 14.33, 12.76 ppm.

**5-(Diethylamino)-2-formylphenyl 4-bromobutanoate:** Diethylaminesalicaldehyde (1g, 5.0 mmol, 1.0 equiv.) was dissolved in 20 mL of acetonitrile (dry) in a 100 mL round bottom flask which was flushed with nitrogen for 15 min. Then, 4-bromobutyric acid (860 mg, 5.0 mmol, 1 equiv.) was added followed by DMAP (630 mg, 1.0 mmol, 0.2 equiv.). EDC (1.2g, 7.5 mmol, 1.5 equiv.) was added to the reaction mixture and stirred for 24 h at room temperature. The product was purified using column chromatography starting with EtOAc 10%:Hexane 90%, and the desired product was eluted with EtOAc 30%:Hexane 70% yielding 699mg of a yellow oil (40% yield). ^1^H NMR (400 MHz, CDCl_3_): δ 1.22 (m, 6H), 2.32 (m, 2H), 2.86 (t, 2H, *J* = 7.1 Hz), 3.43 (m, 4H), 3.58 (t, 2H, *J* = 6.4 Hz), 6.26 (dd, 1H, *J* = 7.4, 2.5 Hz), 6.55 (dd, 1H, *J* = 8.9, 2.5 Hz), 7.62 (t, 1H, *J* = 6.9 Hz), 9.7 (s, 1H) ppm. ^13^C NMR (100.61 MHz, CDCl_3_): δ 12.65, 27.65, 32.57, 32.93, 44.94, 104.74, 108.52, 116.31, 134.95, 153.25, 168.94, 171.23, 186.90 ppm.


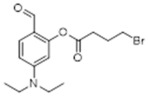


**2-(2-(Benzo[d]oxazol-2-yl)-3-ethoxy-3-oxoprop-1-en-1-yl)-5-(diethylamino)phenyl 4-bromobutanoate, Compound 3:** 5-(Diethylamino)-2-formylphenyl 4-bromobutanoate (699 mg, 2.0 mmol, 1.0 equiv.) was dissolved in dichloromethne (DCM anh., 10 mL) in a 50 mL round bottom flask. Ethyl 2-(benzo[d]oxazol-2-yl)acetate (**6**, 837 mg, 4.0 mmol, 2.0 equiv.), piperidine (69 mg, 0.81 mmol, 0.4 equiv.), and acetic acid (49 mg, 0.81 mmol, 0.4 equiv.) were then added. The reaction mixture was refluxed overnight. After cooling to room temperature and the removal of the solvent under reduced pressure, the product was purified using preparative HPLC using an acetonitrile 70%:water 30% solvent mixture. Compound **18** was obtained as a reddish-orange oil as a mixture of *Z* and *E* isomers (550 mg, 51% yield, ~2:1 *E*:*Z* mixture).


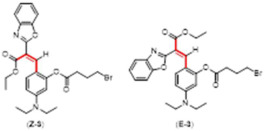


***Z*-isomer:** ^1^H NMR (400 MHz, CD_3_CN): δ = 7.68 (s, 1 H), 7.66 (m, 1 H), 7.59 (m, 1 H), 7.39 (m, 1 H), 7.37 (m, 1 H), 7.36 (m, 1 H), 6.64 (m, 1 H), 6.47 (d, 1 H, *J* = 2.3 Hz), 4.41 (q, 2 H, *J* = 7.1 Hz), 3.61 (t, 2 H, *J* = 6.6 Hz), 3.41 (q, 4 H, *J* = 7.1 Hz), 2.84 (t, 2 H, *J* = 7.2 Hz), 2.28 (m, 2 H), 1.32 (t, 3 H, *J* = 7.1 Hz), 1.15 (t, 6 H, *J* = 7.1 Hz) ppm. ^13^C NMR (100.61 MHz, CD_3_CN): δ = 172.1, 167.5, 162.4, 152.8, 152.7, 151.1, 143.0, 132.0, 130.5, 125.6, 126.9, 120.5, 118.5, 112.2, 111.4, 110.5, 105.68, 62.8, 45.3, 34.0, 33.96, 28.7, 14.4, 12.8 ppm.

***E*-isomer**: ^1^H NMR (400 MHz, CD_3_CN): δ = 8.04 (s, 1 H), 7.76 (m, 1 H), 7.64 (m, 1 H), 7.44 (m, 1 H), 7.43 (m, 1 H), 6.62 (d, 1 H, *J* = 9.1 Hz), 6.39 (d, 1 H, *J* = 2.6 Hz), 6.27 (dd, 1 H, *J* = 9.1 Hz, *J* = 2.6 Hz), 4.23 (q, 2 H, *J* = 7.1 Hz), 3.61 (t, 2 H, *J* = 6.6 Hz), 3.31 (q, 2 H, *J* = 7.1 Hz), 2.79 (t, 2 H, *J* = 7.2 Hz), 2.28 (m, 2 H), 1.23 (t, 3 H, *J* = 7.1 Hz), 1.06 (t, 3 H, *J* = 7.1 Hz) ppm. ^13^C NMR (100.61 MHz, CD_3_CN): δ = 172.1, 166.3, 160.9, 153.7, 152.2, 151.6, 142.2, 142.0, 131.2, 126.7, 125.7, 121.1, 115.2, 111.4, 110.72, 110.3, 105.7, 62.3, 45.33, 34.0, 33.25, 28.70, 14.6, 12.7 ppm.

**6,6′-((1*E*,1′*E*)-hydrazine-1,2diylidenebis(methaneylylidene))bis(3(diethylamino)phenol), Compound 7:** Diethylaminesalicaldehyde (316 mg, 1.62 mmol, 1.0 equiv.) was dissolved in 10 mL acetonitrile in a 20 mL vial. Hydrazine monohydrate (81.0 mg, 48.8 mmol, 30 equiv.) was added to this solution which was stirred at room temperature overnight. The product was purified using column chromatography eluted with DCM affording 115 mg of **7** as an orange solid (18.5% yield). ^1^H NMR (400 MHz, CDCl_3_): δ 1.38 (t, 12H, *J* = 7.1 Hz), 3.57 (q, 8H, *J* = 7.1 Hz), 6.40 (d, 2H, *J* = 2.4 Hz), 6.43 (dd, 2H, *J* = 8.7, 2.5 Hz), 7.27 (m, 2H), 8.63 (S, 2H), 12.00 (S, 2H) ppm. ^13^C NMR (100.61 MHz, CDCl_3_): δ 12.84, 44.69, 97.98, 104.08, 107.06, 133.41, 151.32, 161.03, 161.57 ppm. LC-MS, M+H^+^, *m*/*z* Calc. 382.24, Found 383.30. UV-Vis (λ_abs_ in CH_3_CN, *ε* $3.4\times 10^{4}\;(2)$ M^−1^ cm^−1^): 280 and 415 nm. Fluorescence (λ_em_ in CH_3_CN, *ϕ_F_ =* 0.22 (3)): 481 nm.


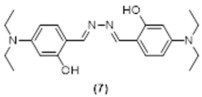


**Diethyl 2-(4-(diethylamino)-2-(propionyloxy)benzylidene)malonate, Compound 8:** 5-(diethylamino)-2-formylphenyl propionate (222 mg, 0.89 mmol, 1.0 equiv.) was dissolved in DCM (anh., 6 mL) in a 50 mL round bottom flask. Diethylmalonate (367 mg, 1.78 mmol, 2.0 equiv.) was added to this solution, followed by piperidine (30.03 mg, 0.35 mmol, 0.4 equiv.) and acetic acid (21.4 mg, 0.35 mmol, 0.4 equiv.). The mixture was refluxed overnight. The desired product was purified using HPLC acetonitrile 70%:water 30%, affording 38 mg of a pure yellow oil (11% yield). ^1^H NMR (400 MHz, CDCl_3_): δ 1.17 (t, 6H, *J* = 7.1 Hz), 1.30 (t, 3H, *J* = 5.3 Hz), 1.31 (t, 6H, *J* = 7.3 Hz), 2.65 (dt, 2H, *J* = 7.5, 5.6 Hz), 3.35 (m, 4H), 4.25 (q, 2H, *J* = 7.1 Hz), 4.33 (q, 2H, *J* = 7.1 Hz), 6.30 (t, 1H, *J* = 5.9 Hz), 6.48 (m, 1H), 7.39 (t, 1H, *J* = 7.1 Hz), 7.71 (s, 1H) ppm. ^13^C NMR (100.61 MHz, CDCl_3_): δ 9.36, 12.70, 14.25, 27.96, 44.71, 61.38, 104.80, 109.28, 112.14, 120.93, 130.17, 135.97, 150.77, 152.25, 165.09, 167.87, 172.71 ppm. LC-MS, M+H^+^, *m*/*z* Calc. 391.46, found 392.20.


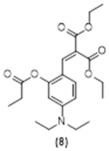


**Ethyl 7-(diethylamino)-2-oxo-2H-chromene-3-carboxylate, Compound 11:** Diethylamino salicylaldehyde (727 mg, 2.48 mmol, 1.0 equiv.) was dissolved in 6 mL of dry DCM in a 50 mL flask. Diethylmalonate (1.02 g, 4.98 mmol, 2.0 equiv.) was added to this solution, followed by piperidine (84 mg, 0.99 mmol, 0.4 equiv.). Acetic acid (58.9 mg, 0.99 mmol, 0.4 equiv.) was then added to the reaction mixture which was refluxed overnight. The completion of the reaction was monitored using TLC. The precipitate was filtered and washed three times with 2-Butanol, dried under reduced pressure to give compound. The product was purified using column chromatography EtOAc 70%:Hexane 30%, affording 215 mg of **11** as a yellow oil (30% yield). ^1^H NMR (400 MHz, CDCl_3_): δ 1.15 (t, 6H, *J* = 7.1 Hz), 1.31 (dd, 3H, *J* = 8.8, 5.4 Hz), 3.36 (q, 4H, *J* = 7.1 Hz), 4.29 (q, 2H, *J* = 7.1 Hz), 6.38 (d, 1H, *J* = 2.4 Hz), 6.52 (dd, 1H, *J* = 9.0, 2.5 Hz), 7.27 (m, 1H), 8.34 (s, 2H) ppm. ^13^C NMR (100.61 MHz, CDCl_3_): δ 12.53, 14.49, 45.20, 61.25, 96.82, 107.78, 109.07, 109.62, 131.13, 149.31, 152.97, 158.42, 158.57, 164.38 ppm. LC-MS, M+H^+^, *m*/*z* Calc. 289.13, Found 290.10. UV-Vis (λ_abs_ in MeCN, *ε* $3.21\times 10^{4}\;(7)$ M^−1^ cm^−1^): 260 and 415 nm. Fluorescence (λ_em_ in MeCN, *ϕ_F_* 0.08 (2)): 464 nm.


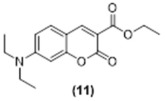


**Diethyl 2-(4-(diethylamino)-2-((4-oxopentanoyl)oxy)benzylidene)malonate, Compound 9:** 5-(diethylamino)-2-formylphenyl 4-oxopentanoate (727 mg, 2.48 mmol, 1.0 equiv.) was dissolved in DCM (anh., 6 mL) in a 50 mL round bottom flask. Diethylmalonate (1.02 g, 4.98 mmol, 2.0 equiv.) was added to this solution, followed by piperidine (84 mg, 0.99 mmol, 0.4 equiv.) and acetic acid (58.9 mg, 0.99 mmol, 0.4 equiv.). The mixture was refluxed overnight. The desired product was purified using column chromatography EtOAc 5%:Hexane 95%, affording 212 mg of a pure yellow oil (19.7% yield). ^1^H NMR (400 MHz, CDCl_3_): δ 1.07 (t, 6H, *J* = 7.1 Hz), 1.21 (q, 6H, *J* = 7.0 Hz), 2.12 (s, 3H), 2.79 (s, 4H), 3.26 (q, 4H, *J* = 7.1 Hz), 4.16 (q, 2H, *J* = 7.1 Hz), 4.23 (q, 2H, *J* = 7.1 Hz), 6.22 (d, 1H, *J* = 2.7 Hz), 6.36 (dd, 1H, *J* = 9.0, 2.6 Hz), 7.27 (m, 1H), 7.60 (s, 1H) ppm. ^13^C NMR (100.61 MHz, CDCl_3_): δ 12.65, 14.22, 28.24, 29.95, 38.11, 44.68, 61.07, 104.75, 109.31, 112.08, 121.05, 130.19, 136.09, 150.77, 152.14, 165.06, 167.81, 171.21, 206.35 ppm. LC-MS, M+H^+^, *m*/*z* Calc. 433.21, Found 434.20.


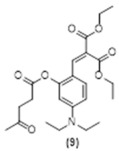


**Diethyl 2-(2-((4-bromobutanoyl)oxy)-4-(diethylamino)benzylidene)malonate, Compound 10:** 5-(diethylamino)-2-formylphenyl 4-bromobutanoate (216 mg, 0.62 mmol, 1.0 equiv.) was dissolved in DCM (anh., 6 mL) in a 50 mL round bottom flask. Diethylmalonate (259 mg, 1.25 mmol, 2.0 equiv.) was added to this solution, followed by piperidine (21.3 mg, 0.25 mmol, 0.4 equiv.) and acetic acid (15.08 mg, 0.35 mmol, 0.4 equiv.). The mixture was refluxed overnight. The desired product was purified using HPLC acetonitrile 70%:water 30%, affording 51.6 mg of a pure yellow oil (17% yield). ^1^H NMR (400 MHz, CDCl_3_): δ 1.17 (t, 6H, *J* = 7.1 Hz), 1.30 (t, 3H, *J* = 5.3 Hz), 2.25 (m, 1H), 2.33 (m, 1H), 2.83 (t, 2H, *J* = 7.3 Hz), 3.38 (dq, 4H, *J* = 21.3, 7.1 Hz), 3.56 (m, 1H), 3.68 (m, 1H), 4.28 (m, 2H), 4.33 (m, 2H), 6.30 (m, 1H), 6.47 (m, 1H), 7.38 (d, 1H, *J* = 9.0 Hz), 7.66 (s, 1H) ppm. ^13^C NMR (100.61 MHz, CDCl_3_): δ 12.99, 14.56, 27.92, 31.93, 33.00, 44.73, 61.74, 104.98, 109.72, 112.38, 121.51, 130.18, 136.18, 151.08, 152.32, 165.34, 168.08, 171.28 ppm. LC-MS, M+H^+^, *m*/*z* Calc. 484.38, Found 484.20.


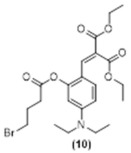


## 4. Conclusions

Building on our recent studies with a pre-coumarin OFF–ON sensor for fluoride anions [19], we decided to apply the same strategy towards the generation of hydrazine sensors. To that end, we adapted three known phenol protecting groups to a pre-coumarin scaffold. As expected, our probes reacted with hydrazine generating a fluorescent signal. The limits of detection in the low μM range were calculated for all three compounds. These LOD values are comparable to other previously reported sensors for hydrazine [36,37], which could suggest that the reaction with hydrazine remains as the rate limiting step of the sensing process. Interestingly, a study of the sensing mechanism revealed that in our case the hydrazine deprotection may be outperformed by a retro-Knoevenagel reaction, where excess hydrazine acts as a nucleophile and a base. Formally, two moles of 4-(diethylamino)-2-hydroxybenzaldehyde react with one mol of hydrazine yielding the fluorescent diimide **7**. While evaluating the selectivity of our hydrazine sensors we discovered that, in contrast to previous reports, our pre-coumarins also react with primary amines (e.g., ethylamine), generating a fluorescent signal that corresponds to the phenol deprotection followed by cyclization to the expected coumarin **4**. Here, we establish the potential of compound **3** as a theranostic turn-on coumarin precursor (for a recent review on “Activity-Based Fluorescence Diagnostics for Cancer”, see ref. [50]). The selective reaction of **3** with ALDOA blocks the nucleophilic amine in the binding site of the enzyme, while the cleavage of the ester group in pre-coumarin **3** induces the formation of the fluorescent coumarin **4**. The inhibition of the enzyme is proportional to the detected fluorescence signal. In principle, this theranostic process could enable the therapeutic inhibition of ALDOA in cancer cells and its concomitant quantitative diagnostic detection by tracking the fluorescence response. The biological tests to confirm this hypothesis and the optimization of the pre-coumarin probe are a work in progress.

## Data Availability

Data is contained within the article.

## Supplementary Materials

The following supporting information can be downloaded at: https://www.mdpi.com/article/10.3390/molecules29102175/s1, Figures S1–S40: NMR experiments; Figures S41–S45: Proposed reaction mechanisms. Figures S46–S60: UV-vis and Fluorescence Results. Figure S61: Stacked and zoomed in, 1H NMR spectra of 4, and 2 upon addition of 0, 1.0, 15.0 and 30.0 equiv. N_2_H_4_.
