# Supplementary material for: Turn-on Coumarin Precursor: From Hydrazine Sensor to Covalent Inhibition and Fluorescence Detection of Rabbit Muscle Aldolase
Source: Molecules. 2024 May 7;29(10):2175. doi: 10.3390/molecules29102175 (PMC11123778; doi:10.3390/molecules29102175)
Supplement: Supplementary file 1 [file molecules-29-02175-s001.zip › molecules-2982870-supplementary.pdf]

Electronic Supporting Information for

Turn-On Coumarin Precursor: From Hydrazine Sensor to Covalent Inhibition and Fluorescence Detection of Rabbit Muscle Aldolase

Sara Amer,[a] Uri Miles,[a,c] Michael Firer,[b] and Flavio Grynszpan[a]*

TABLE OF CONTENTS

NMR experiments…..……………………………………………….……………….. S2

Reaction Mechanisms …....……………………………………………………... S24

UV-vis and Fluorescence Results…………………………………………………S27

LOD studies…………………………………………………………………….S30

Comparison of a real sample of 4 to the product of the reaction between 2 and hydrazine, an NMR study………………………………………………………...………...S34

NMR experiments

Ethyl 2-(benzo[d]oxazol-2-yl)acetate:

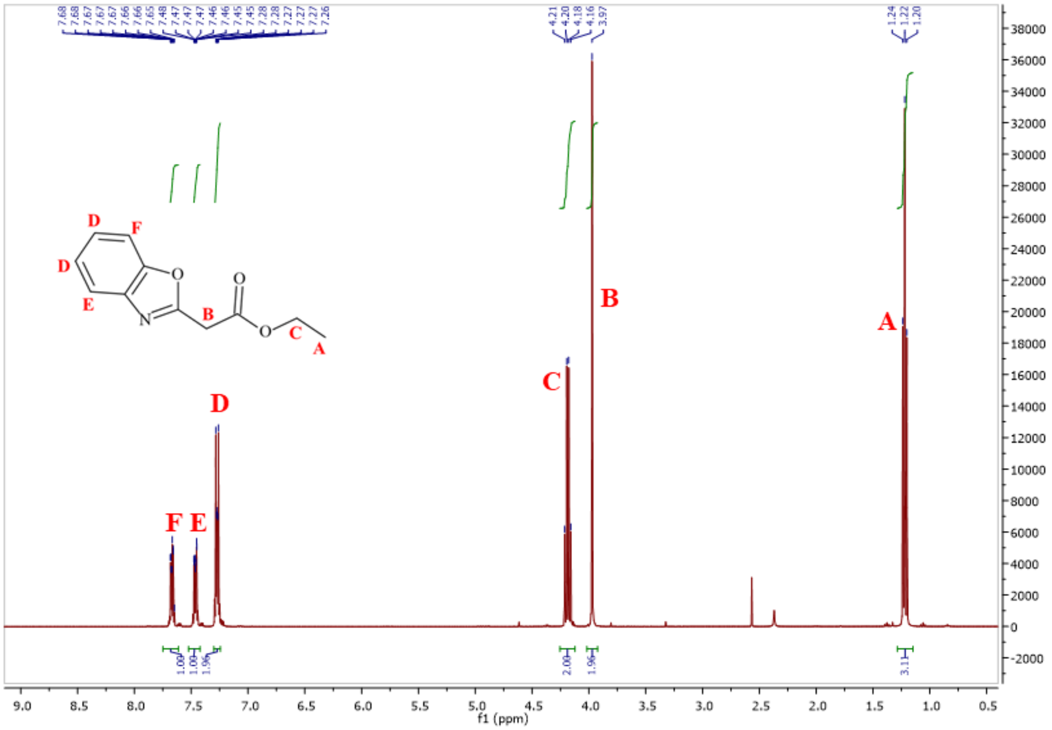

Figure S1: ^{1}H NMR ($CDCl_3$, 300 K) for compound **6**.

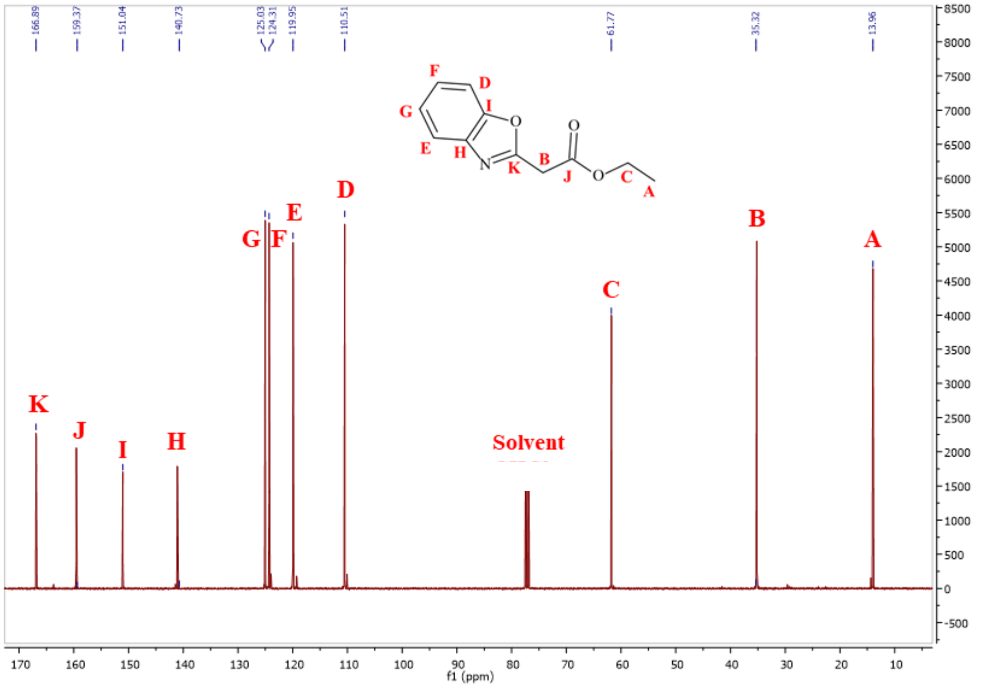

Figure S2: ^{13}C NMR ($CDCl_3$, 300 K) for compound **6**.

3-(Benzo[d]oxazol-2-yl)-7-(diethylamino)-2H-chromen-2-one:

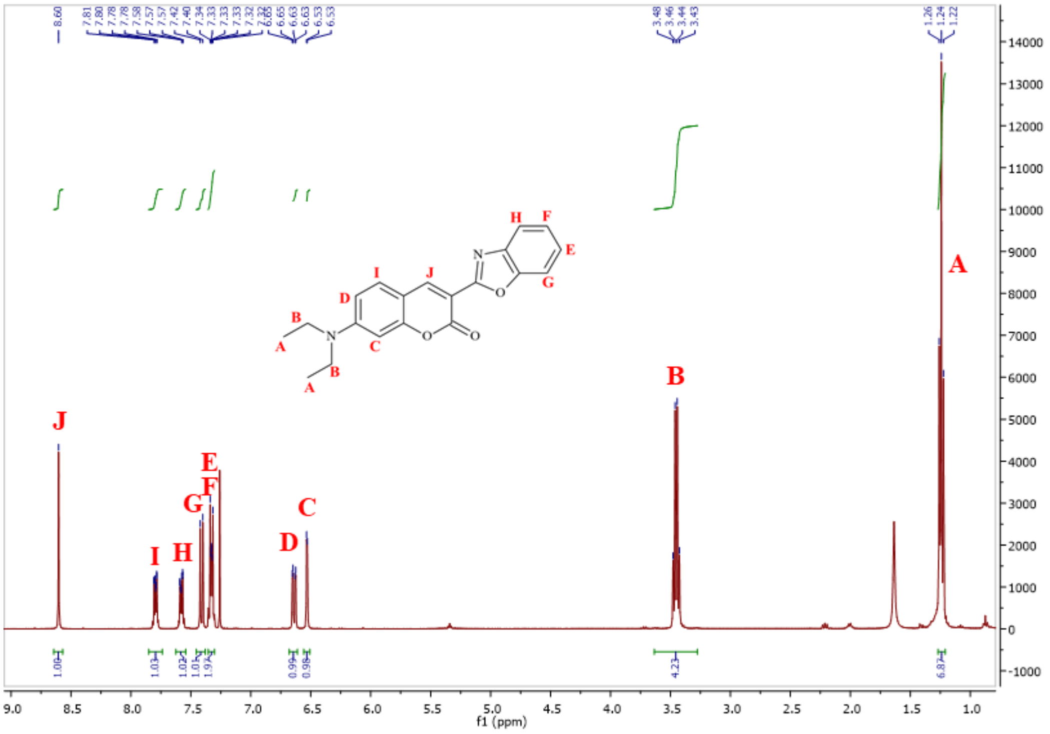

Figure S3: ^{1}H NMR ($CDCl_3$, 300 K) for compound **4**.

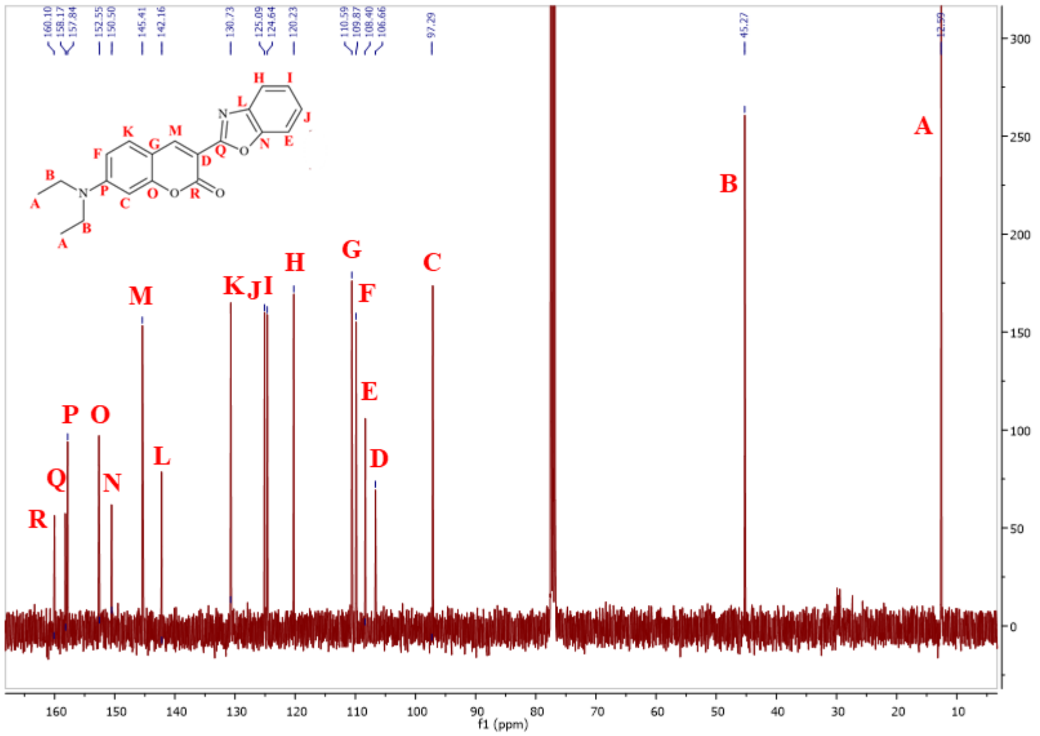

Figure S4: 13C NMR ($CDCl_3$, 300 K) for compound **4**.

5-(Diethylamino)-2-formylphenyl propionate:

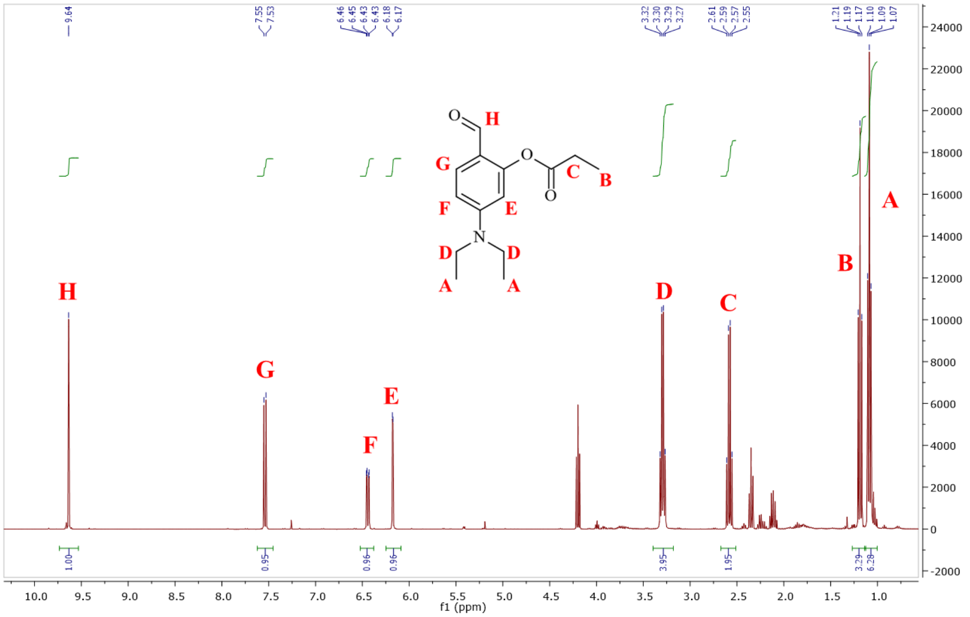

Figure S5: ^{1}H NMR ($CDCl_3$, 300 K) for compound **5**.

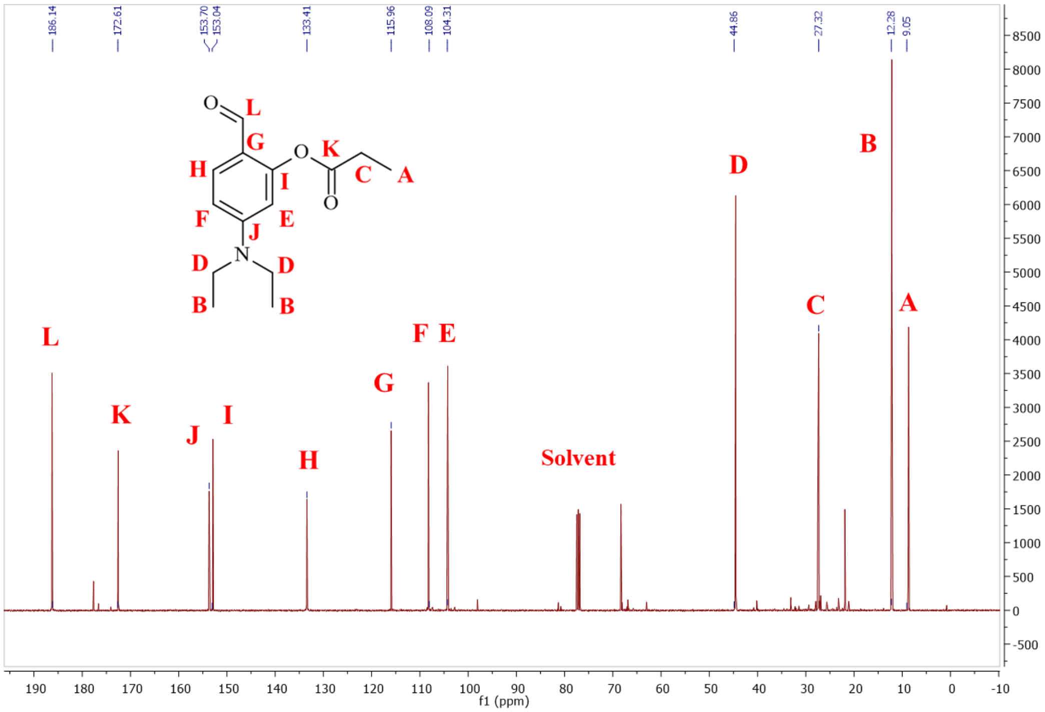

Figure S6: ^{13}C NMR ($CDCl_3$, 300 K) for compound **5**.

(*E*+*Z*) Ethyl-2-(benzo[d]oxazol-2-yl)-3-(4-(diethylamino)-2-(propionyloxy)phenyl)acrylate:

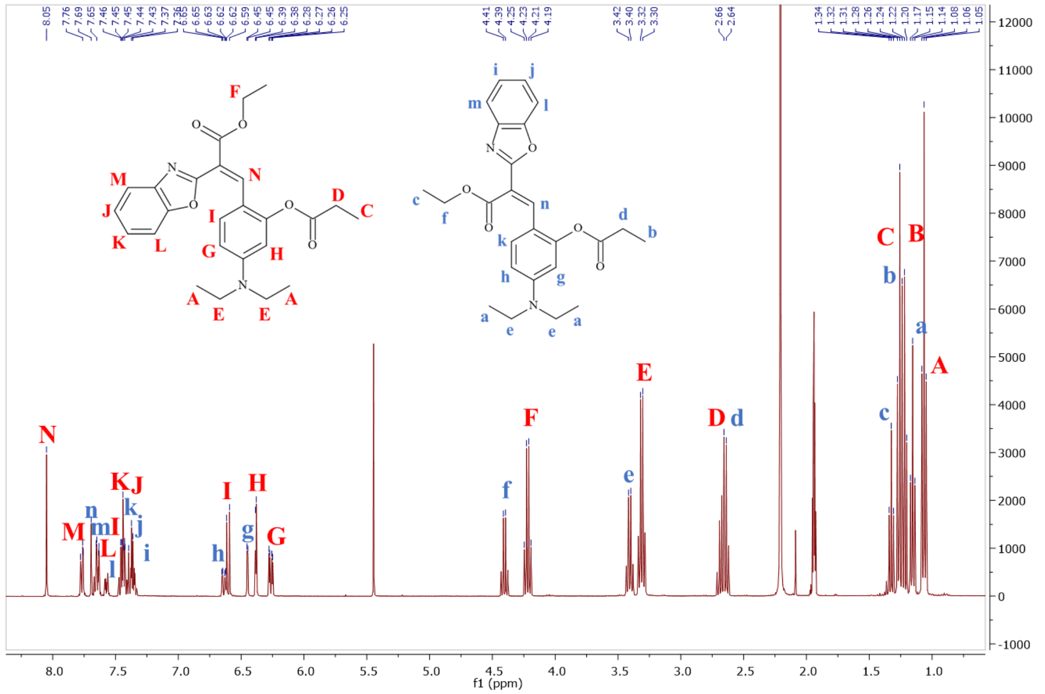

Figure S7: ^{1}H NMR (CD_3CN, 300 K) for compound **1**.

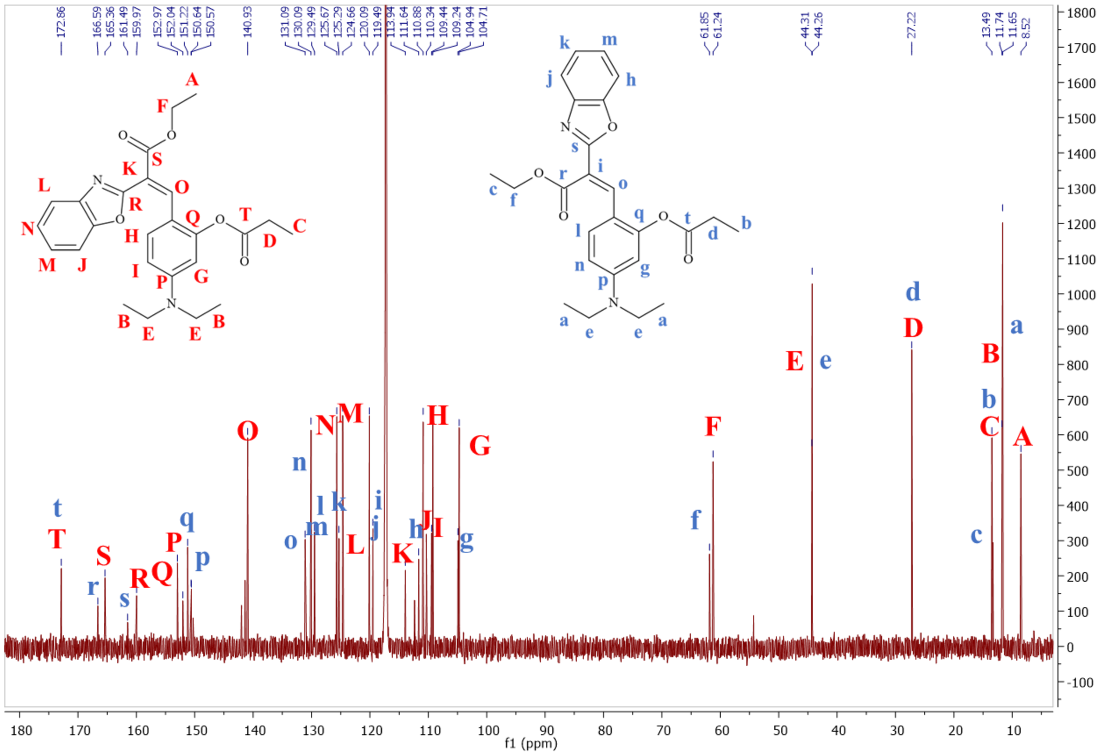

Figure S8: ^{13}C NMR (CD_3CN, 300 K) for compound **1**.

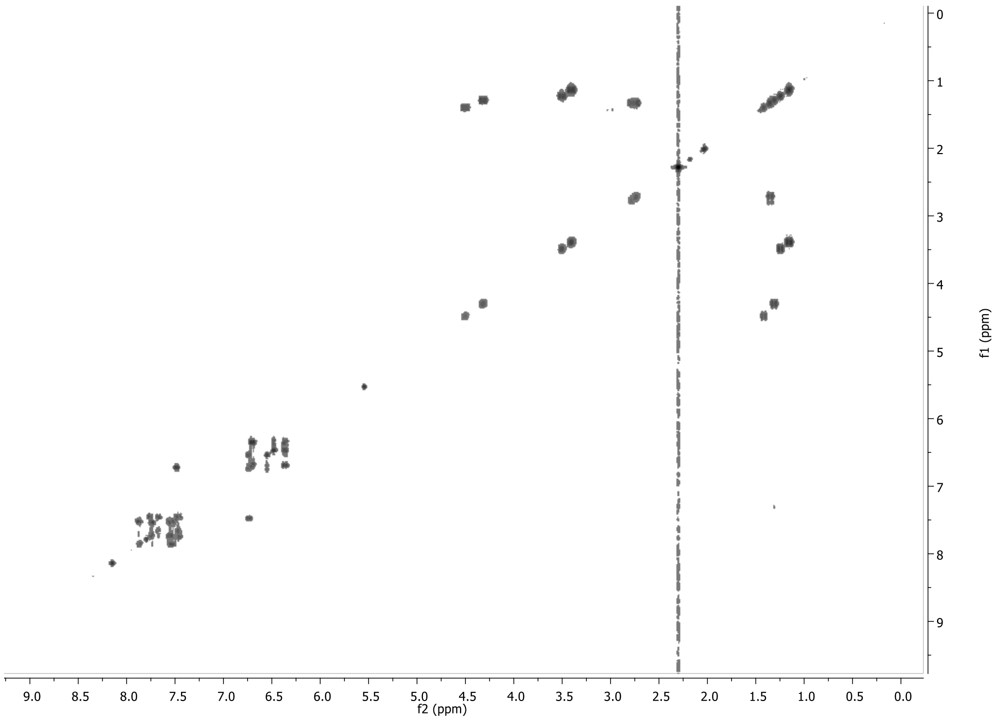

Figure S9: COSY NMR (CD_3CN, 300 K) for compound **1**.

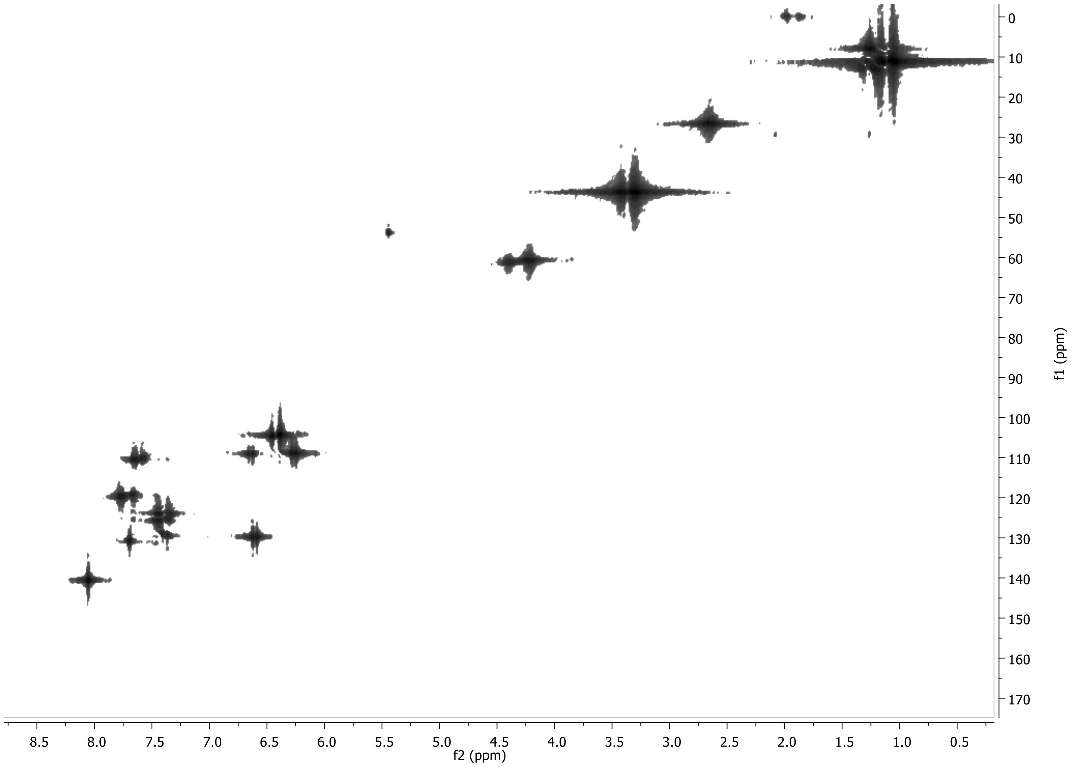

Figure S10: HMQC NMR (CD_3CN, 300 K) for compound **1**.

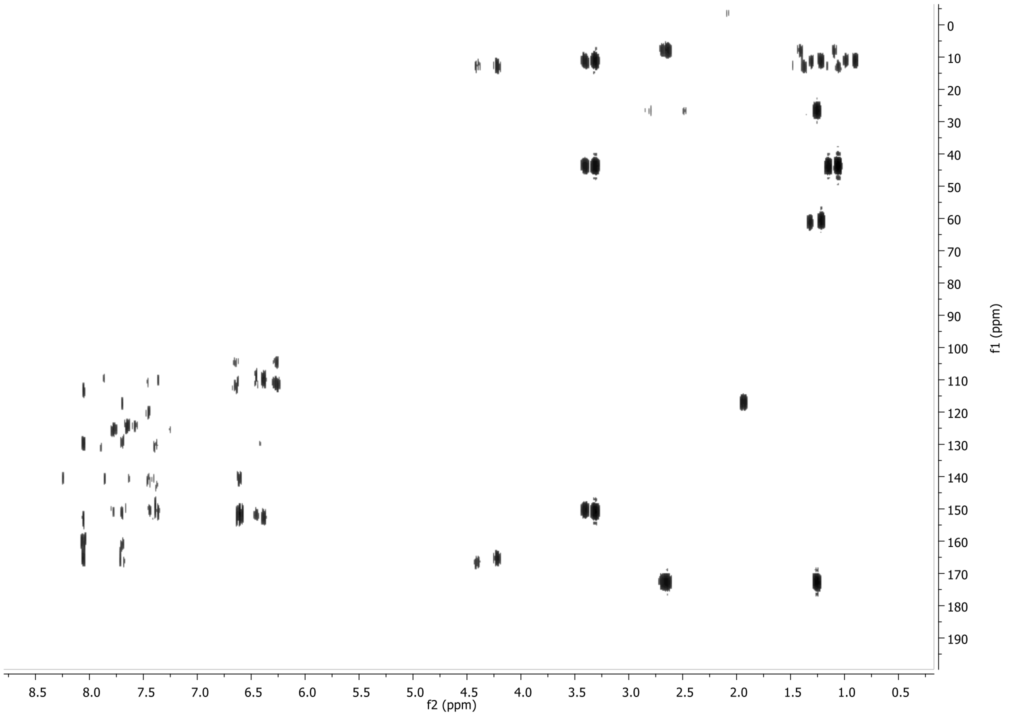

Figure S11: HMBC NMR (CD_3CN, 300 K) for compound **1**.

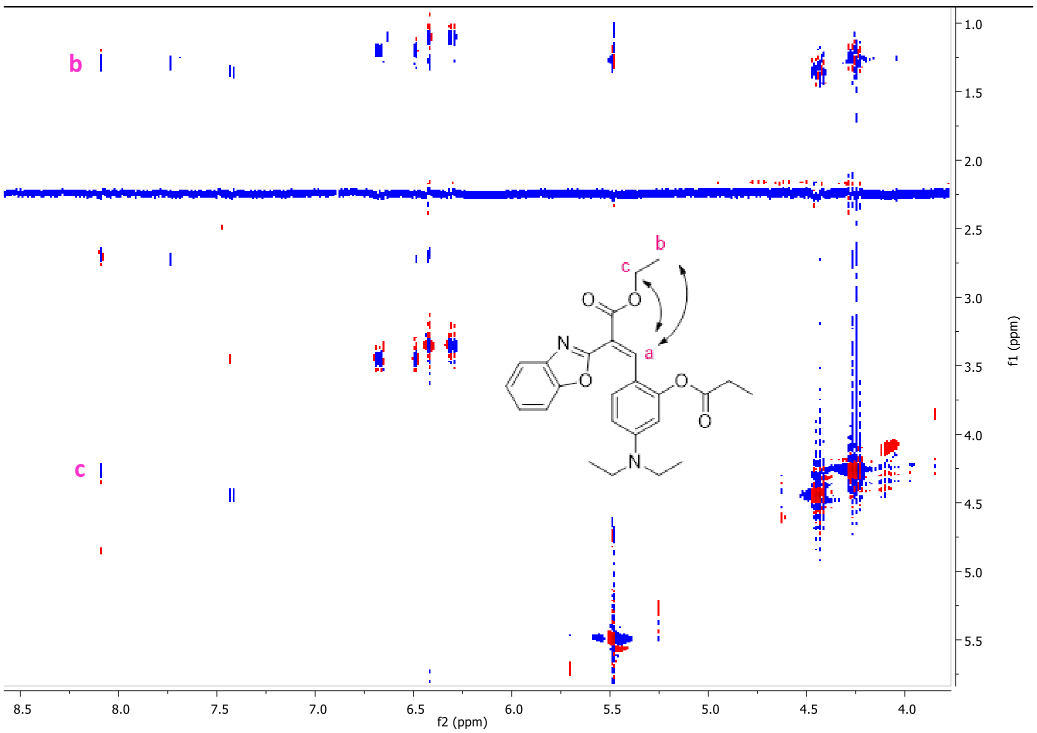

Figure S12: Zoomed in NOESY NMR (CD_3CN, 300 K) for compound **1**.

5-(Diethylamino)-2-formylphenyl 4-oxopentanoate:

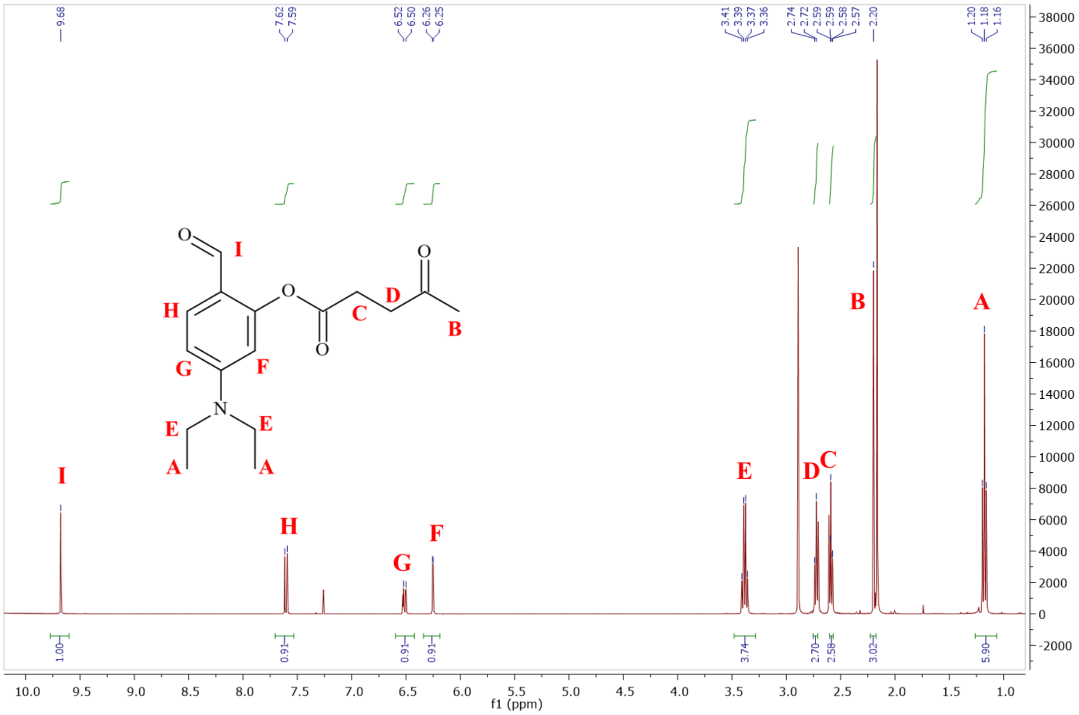

Figure S13: ^{1}H NMR ($CDCl_3$, 300 K) for 5-(Diethylamino)-2-formylphenyl 4-oxopentanoate.

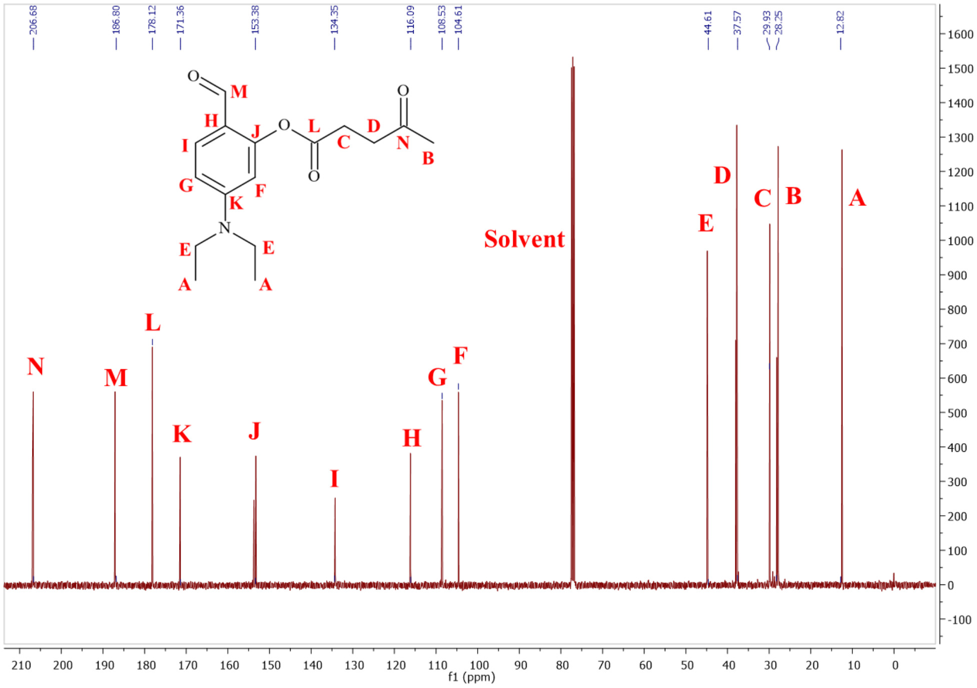

Figure S14: ^{13}C NMR ($CDCl_3$, 300 K) for 5-(Diethylamino)-2-formylphenyl 4-oxopentanoate.

2-(2-(Benzo[d]oxazol-2-yl)-3-ethoxy-3-oxoprop-1-en-1-yl)-5-(diethylamino)phenyl 4-oxopentanoate, Compound 2:

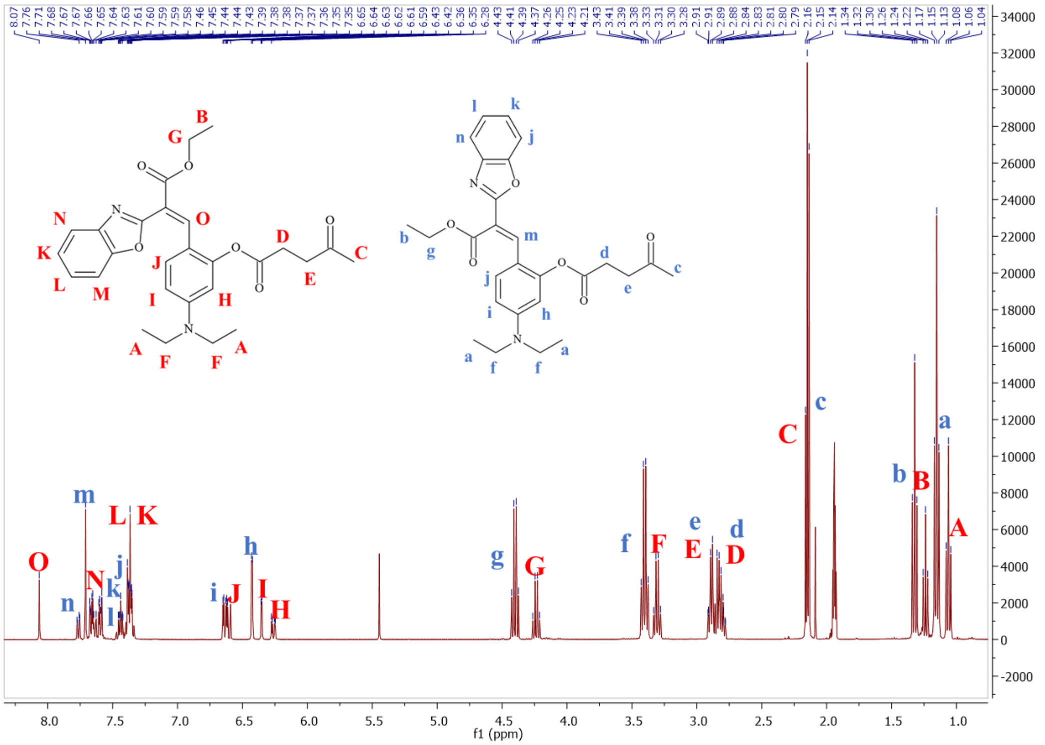

Figure S15: ^{1}H NMR (CD_3CN, 300 K) for compound **2**.

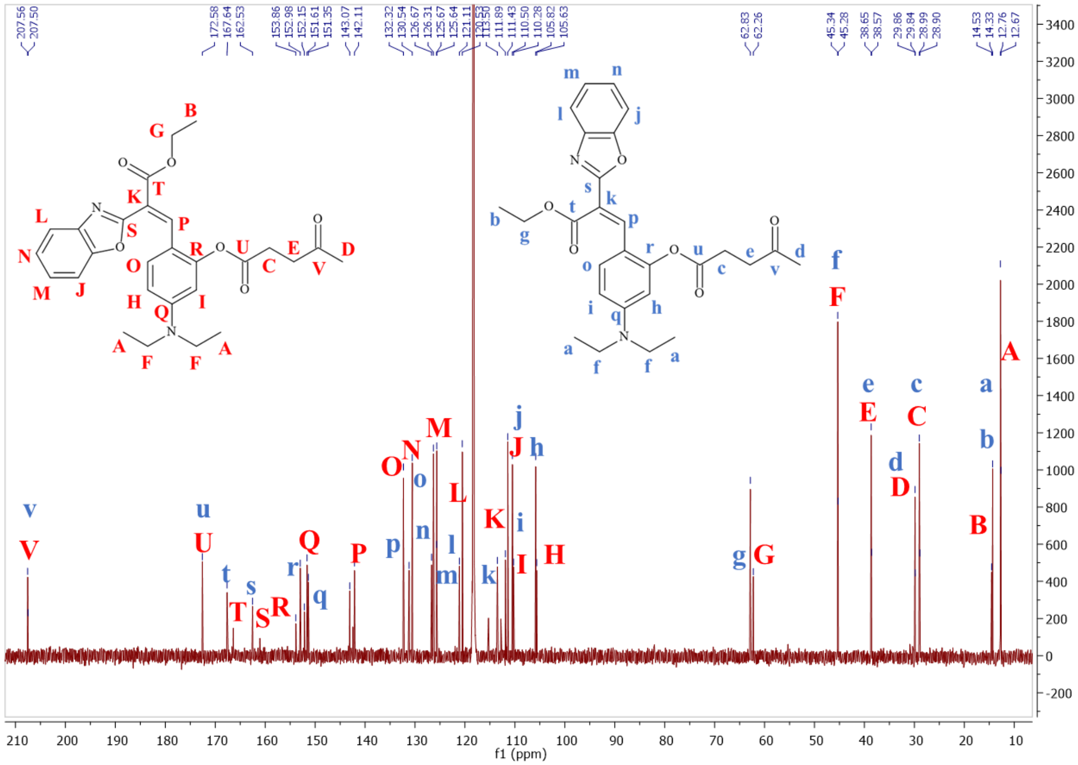

Figure S16: ^{13}C NMR (CD_3CN, 300 K) for compound **2**.

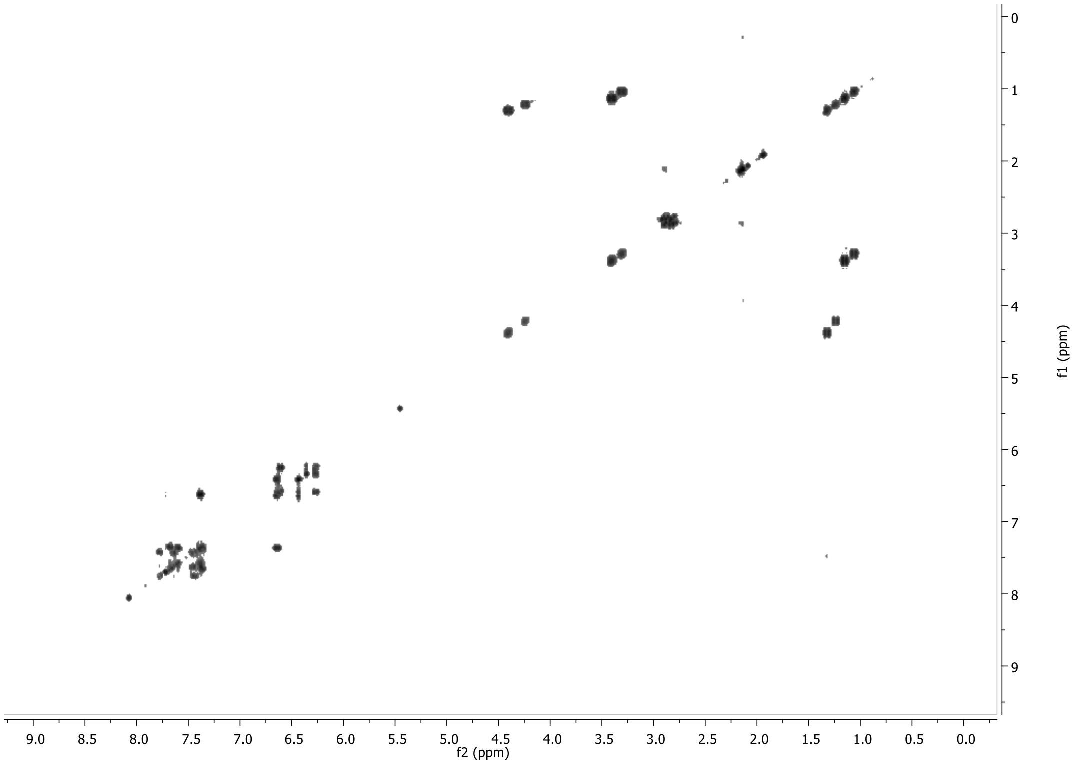

Figure S17: COSY NMR (CD_3CN, 300 K) for compound **2**.

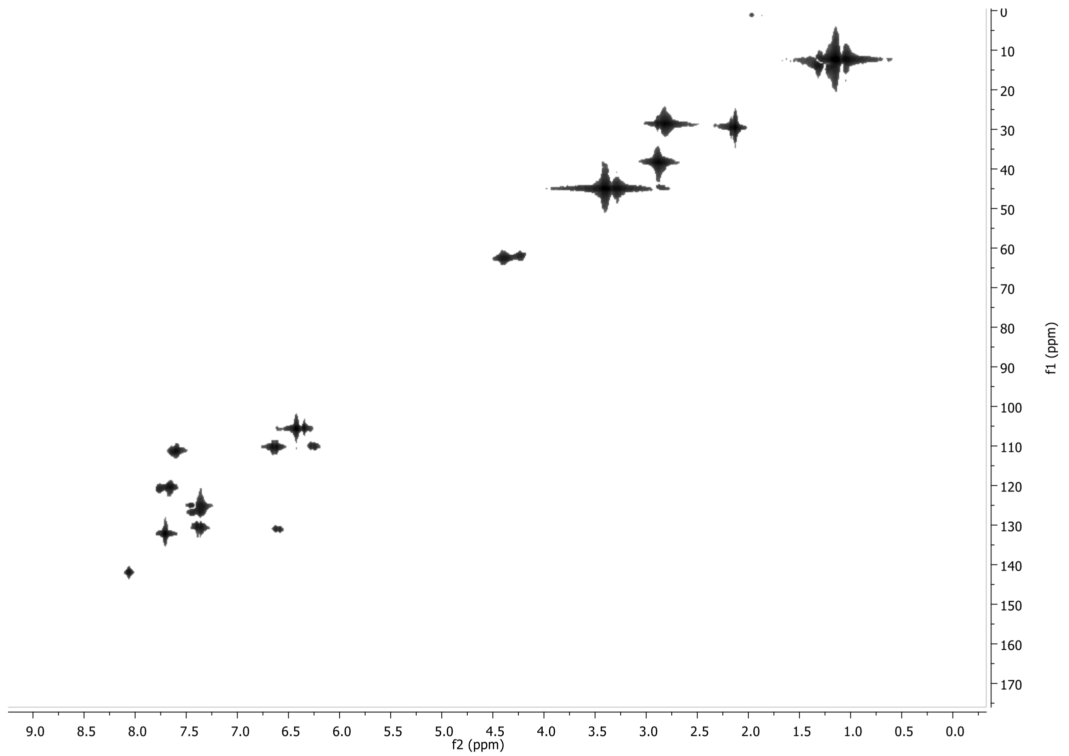

Figure S18: HMQC NMR (CD_3CN, 300 K) for compound **2**.

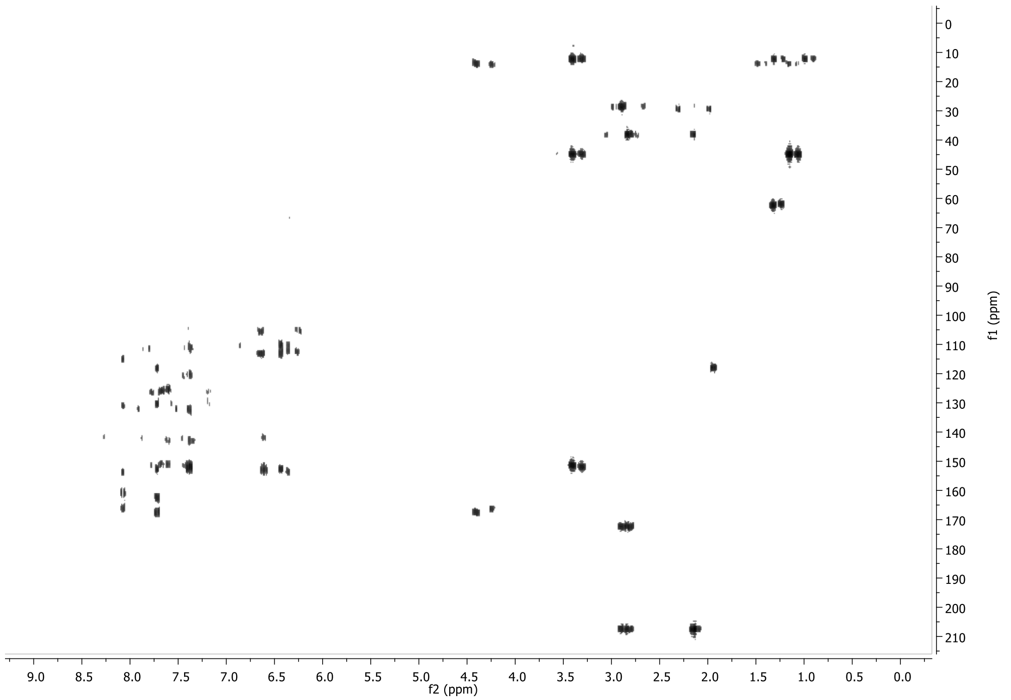

Figure S19: HMBC NMR (CD_3CN, 300 K) for compound **2**.

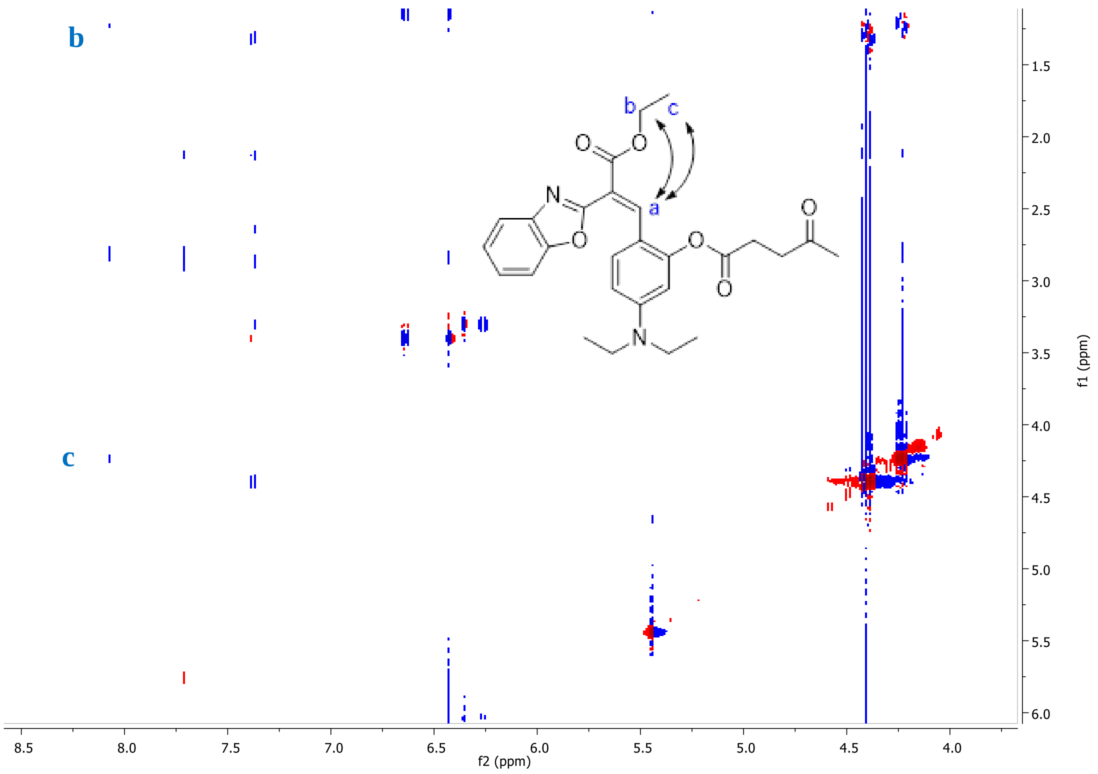

Figure S20: Zoomed in NOESY NMR (CD_3CN, 300 K) for compound **2**.

5-(Diethylamino)-2-formylphenyl 4-bromobutanoate:

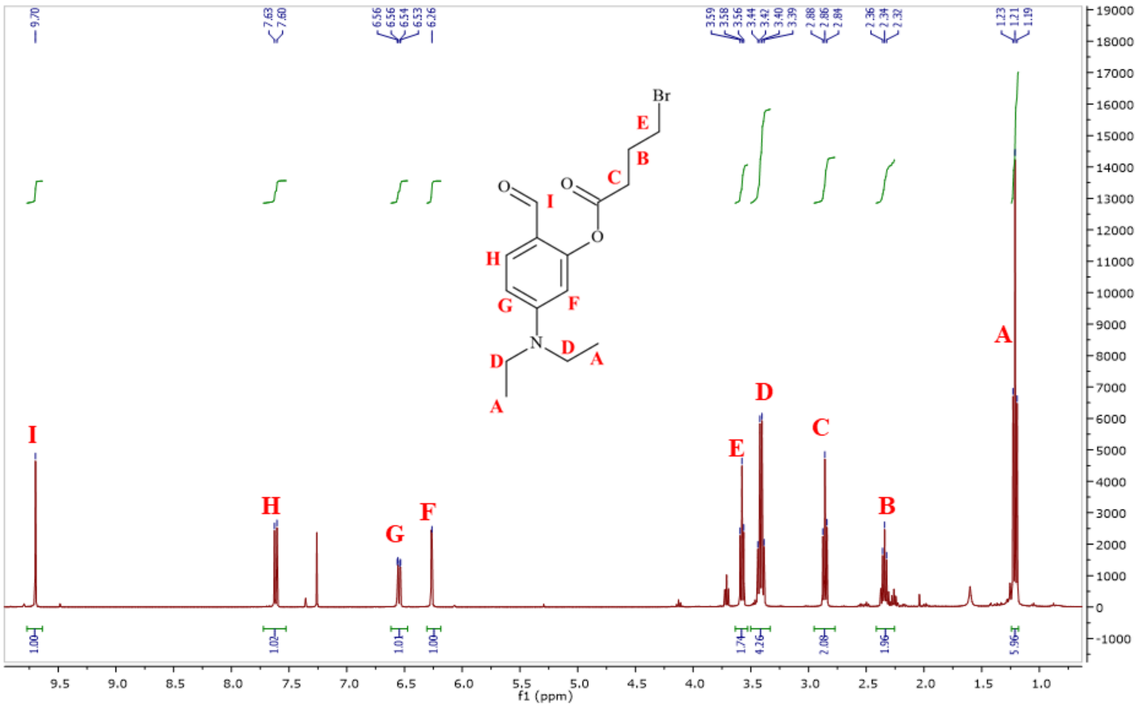

Figure S21: ^{1}H NMR (CDCl3, 300 K) for compound **3**.

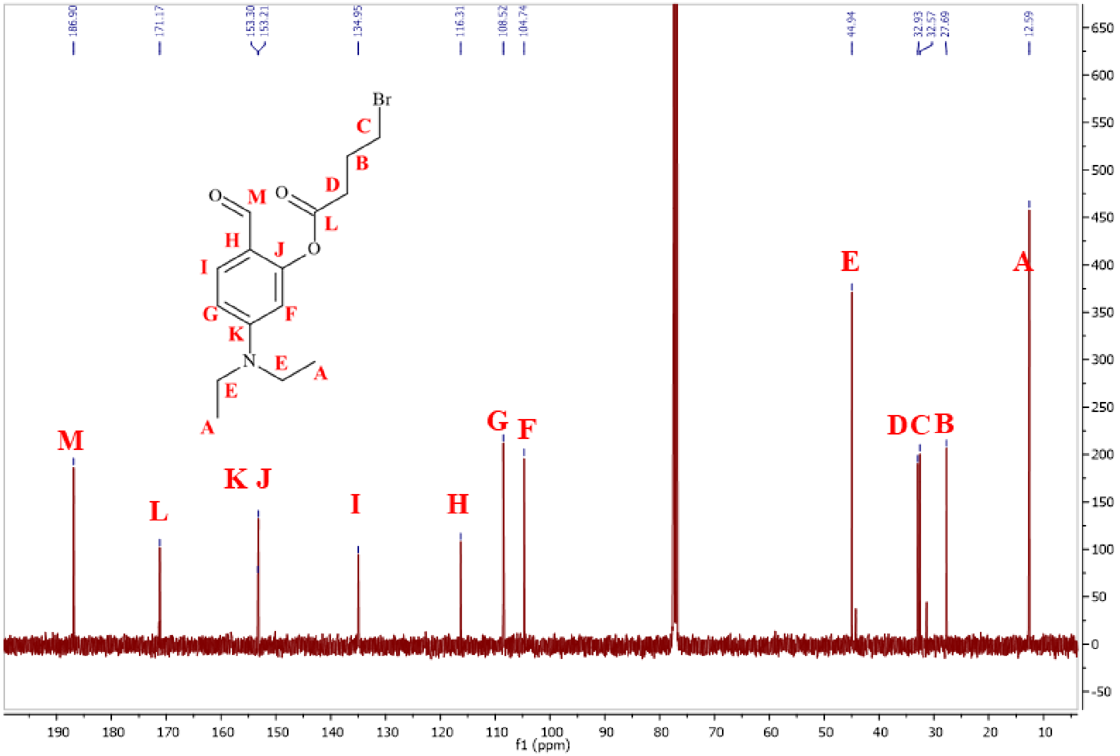

Figure S22: ^{13}C NMR (CDCl3, 300 K) for compound **3**.

2-(2-(Benzo[d]oxazol-2-yl)-3-ethoxy-3-oxoprop-1-en-1-yl)-5-(diethylamino)phenyl 4-bromobutanoate, Compound 3:

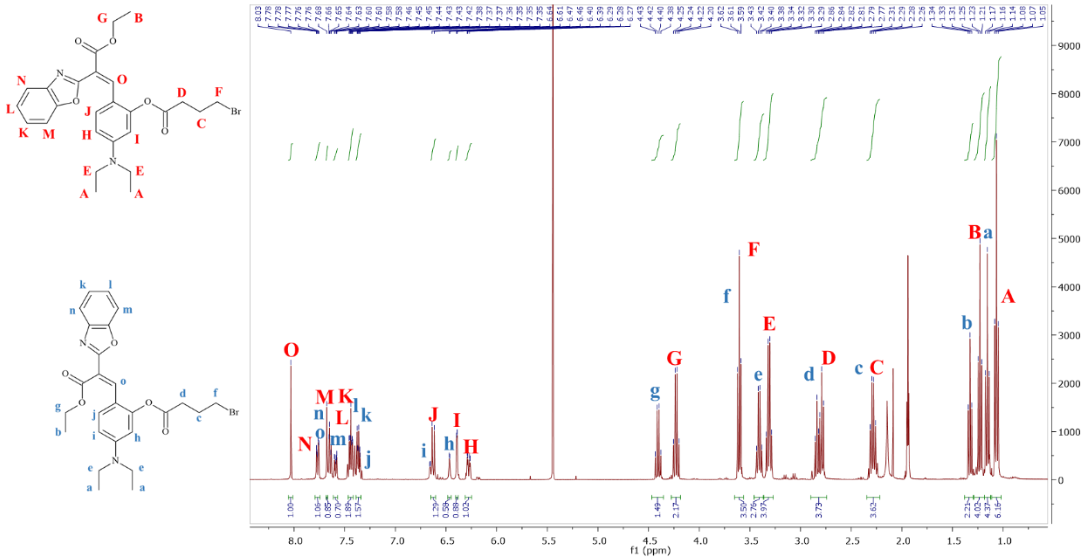

Figure S22: ^{1}H NMR (CD_3CN, 300K) for compound **3** (mixture of *E*:*Z* isomers).

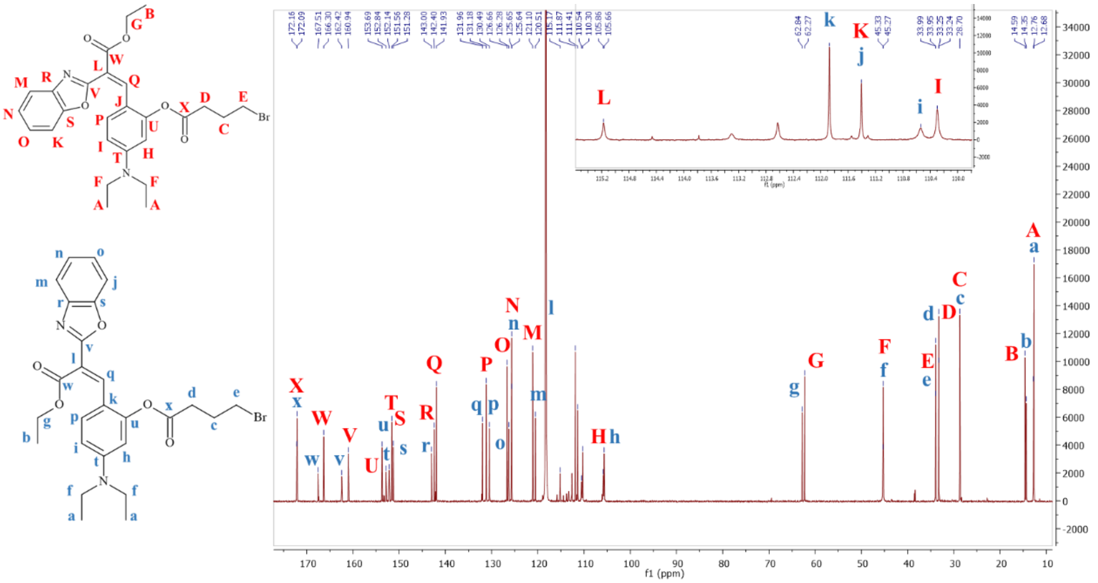

Figure S23: ^{13}C NMR (CD_3CN, 300K) for compound **3** (mixture of *E*:*Z* isomers).

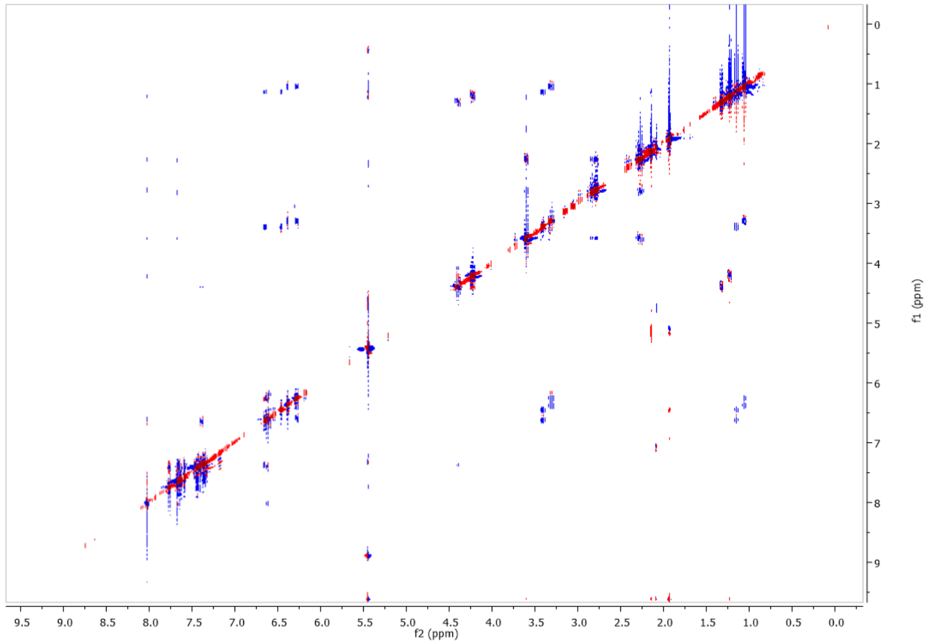

Figure S24: NOESY NMR (CD_3CN, 300 K) for compound **3** (mixture of *Z* and *E* isomers).

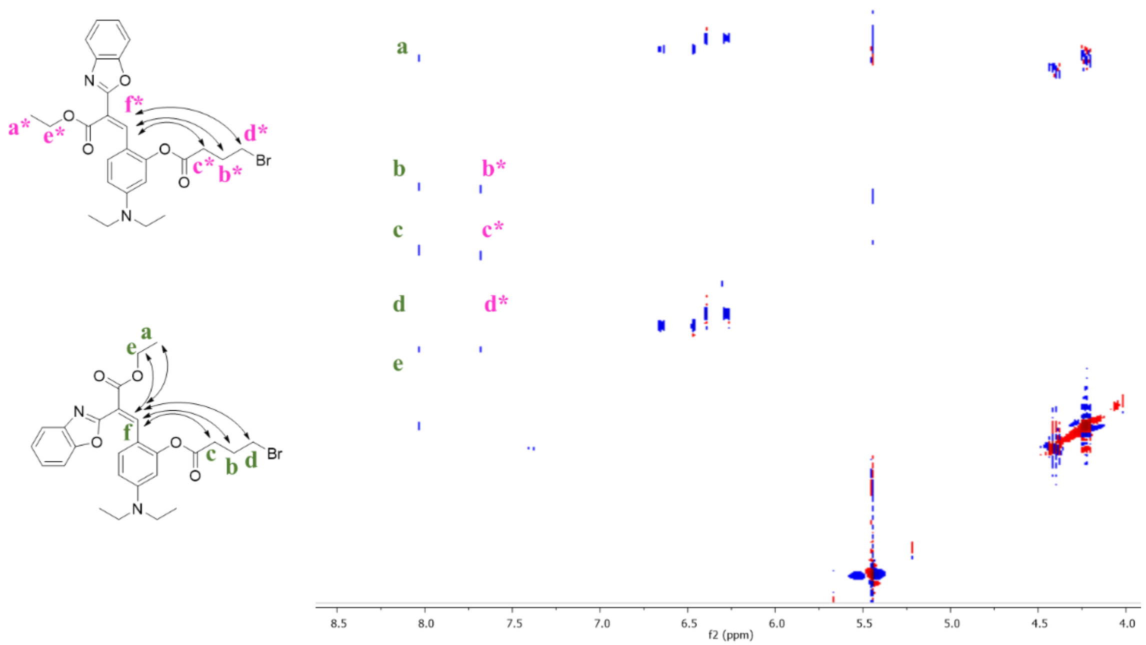

Figure S25: Zoomed-in NOESY NMR (CD_3CN, 300 K) for compound **3** (mixture of *Z* and *E* isomers).

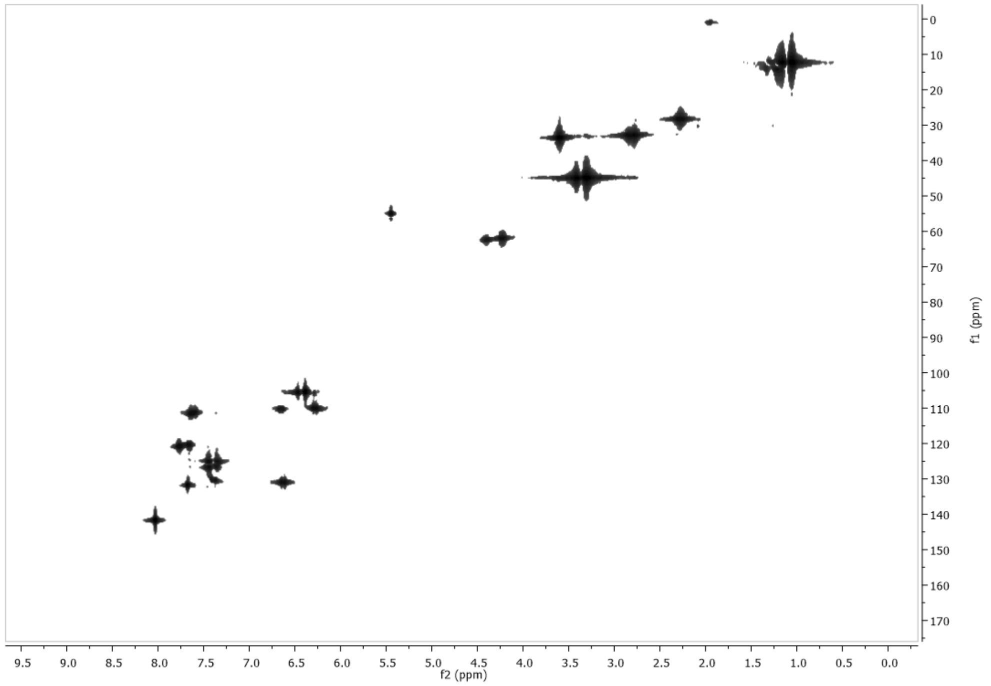

Figure S26: HMQC NMR (CD_3CN, 300 K) for compound **3** (mixture of *Z* and *E* isomers).

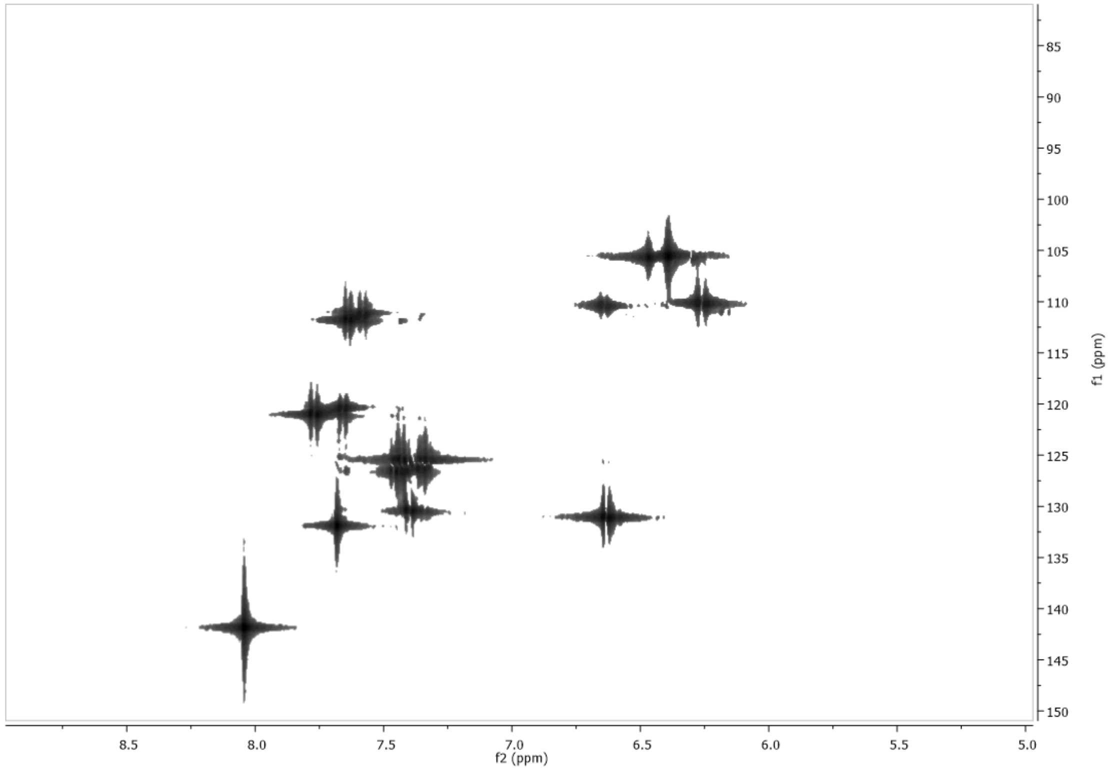

Figure S27: Short-range HMQC NMR (CD_3CN, 300 K) of compound **3** (mixture of *Z* and *E* isomers).

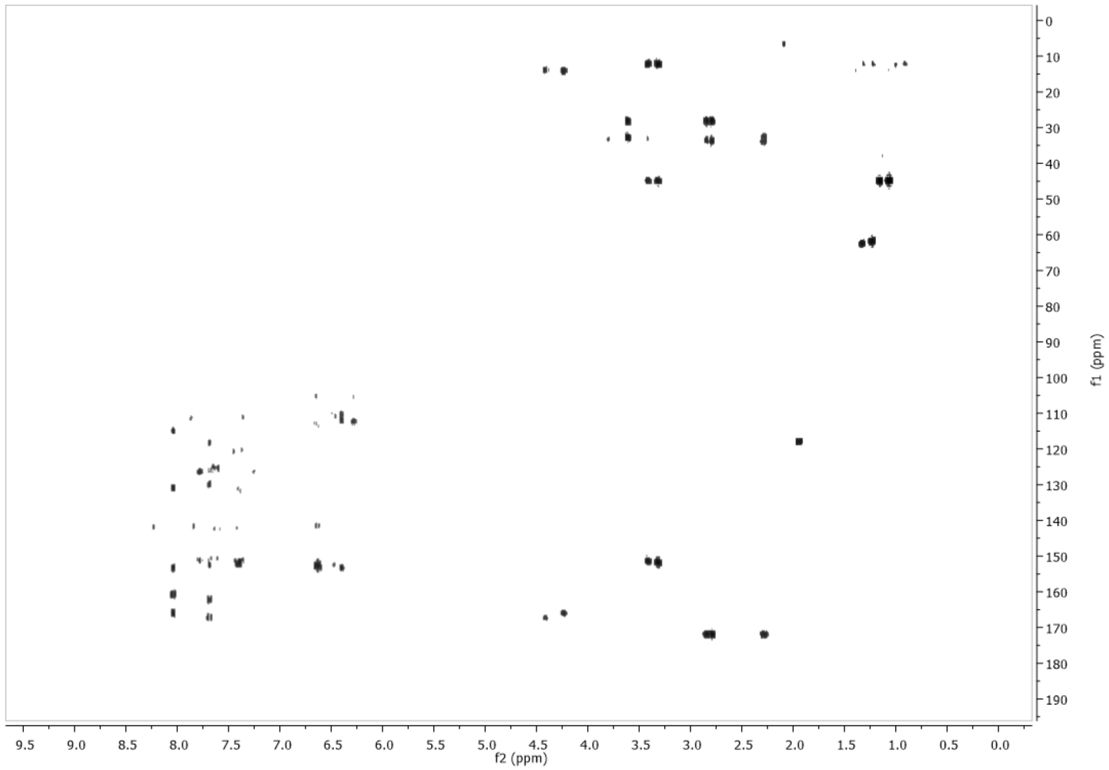

Figure S28: HMBC NMR (CD_3CN, 300 K) for compound **3** (mixture of *Z* and *E* isomers).

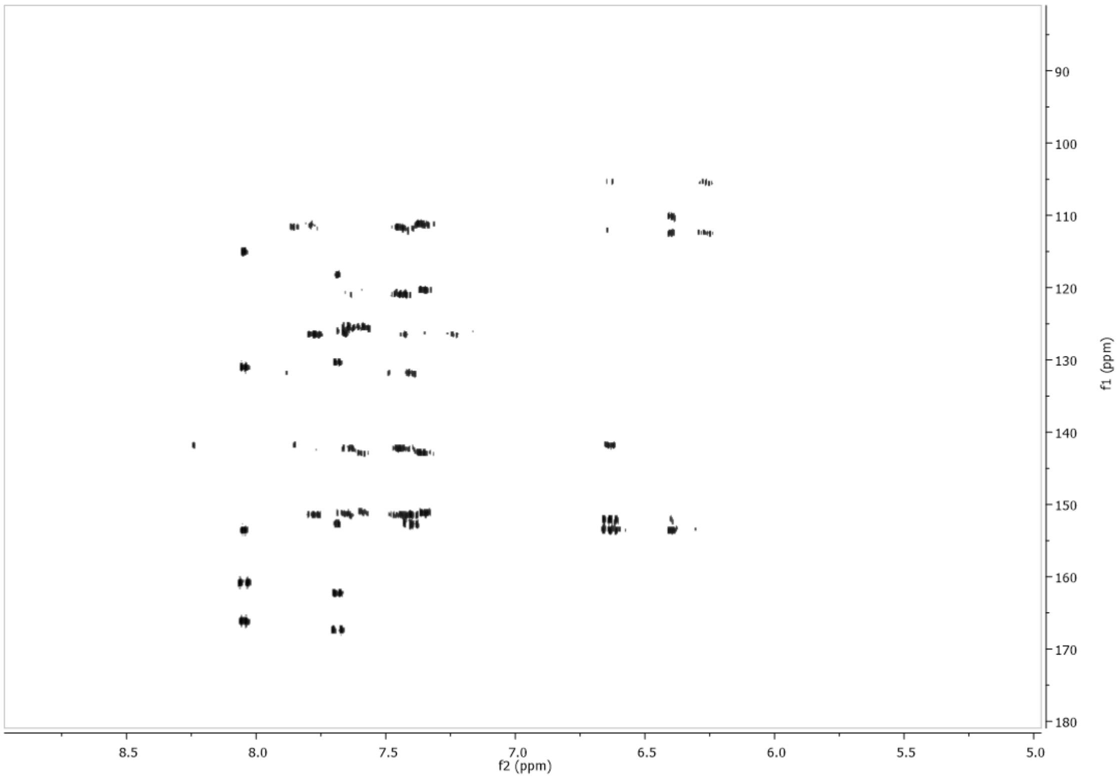

Figure S29: Short-range HMBC NMR (CD_3CN, 300 K) for compound **3** (mixture of *Z* and *E* isomers).

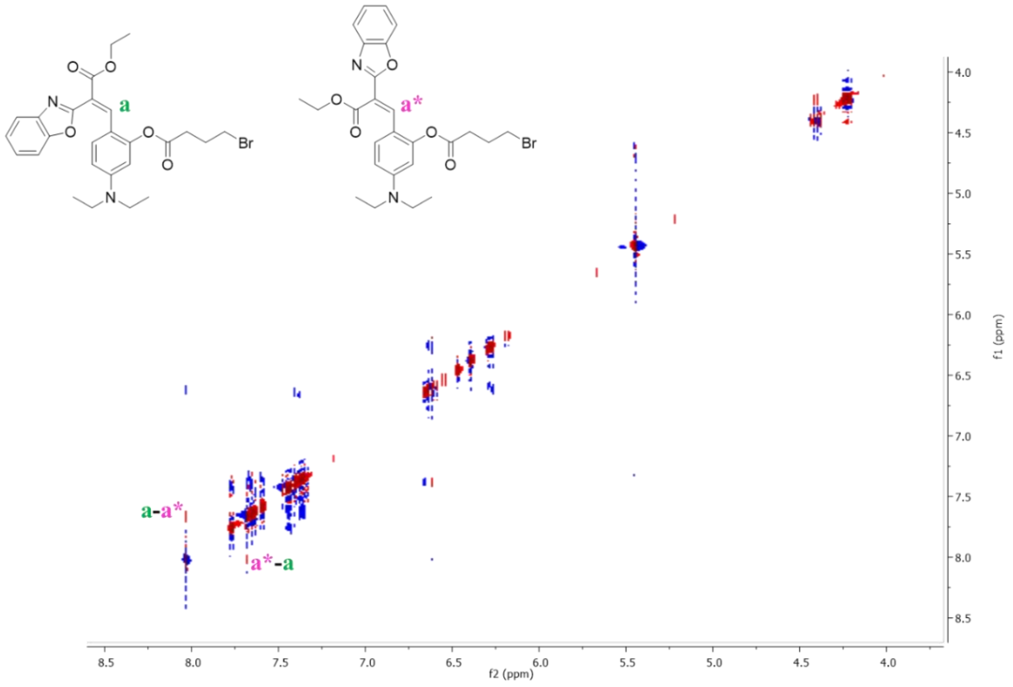

Figure S30: Zoomed-in EXSY experiment (CD_3CN, 300 K) for compound **3** (mixture of *Z* and *E* isomers).

6,6'-((1*E*,1'*E*)-hydrazine-1,2diylidenebis(methaneylylidene))bis(3(diethylamino)phenol), Compound 7:

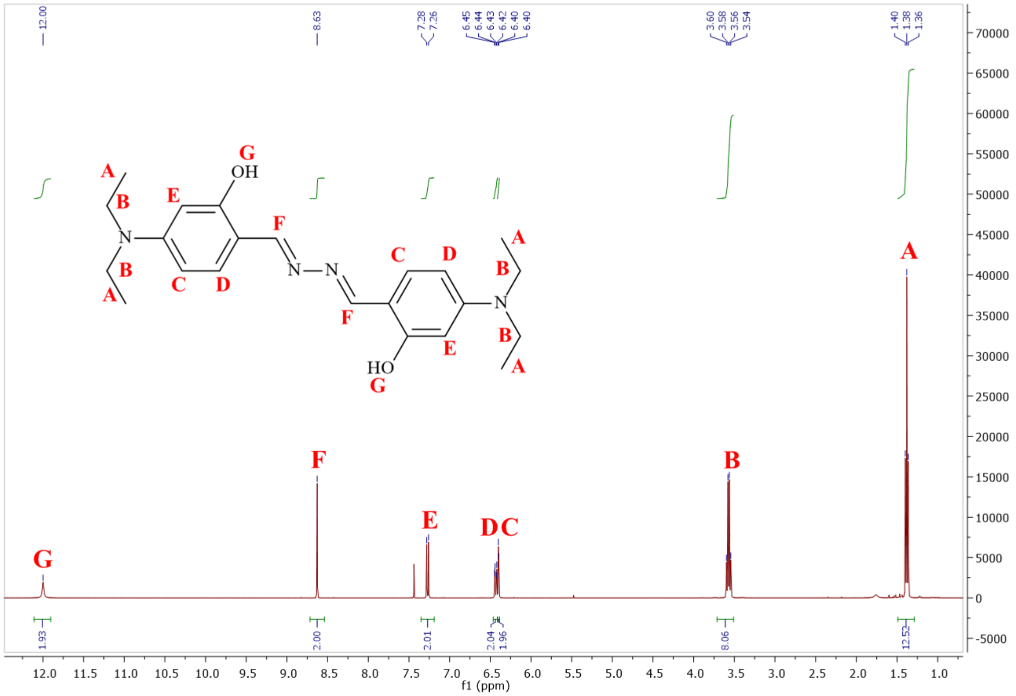

Figure S31: ^{1}H NMR ($CDCl_3$, 300 K) for compound **7**.

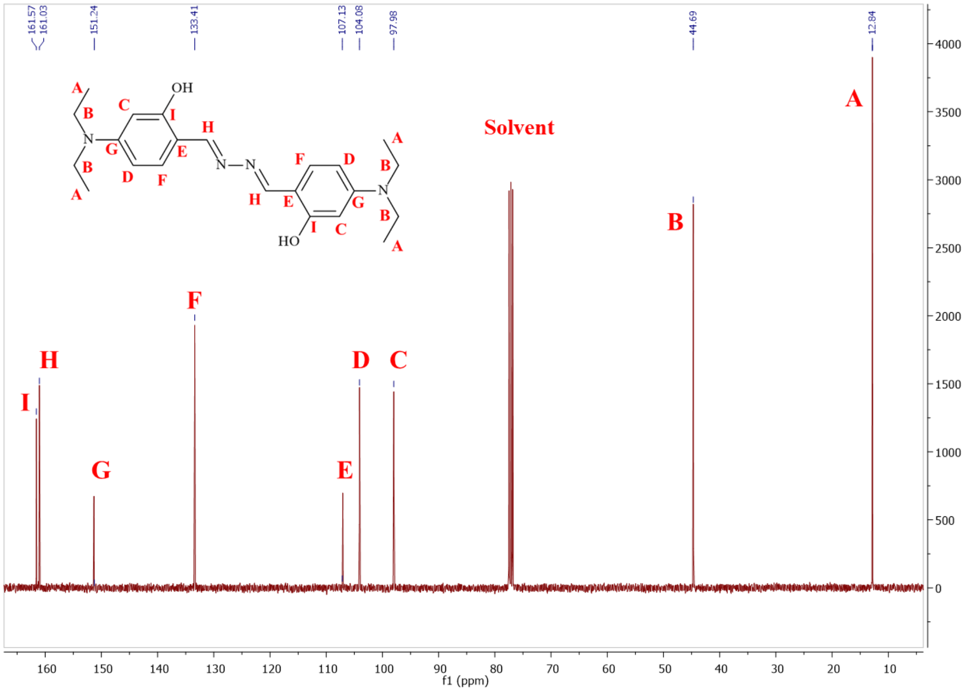

Figure S32: ^{13}C NMR ($CDCl_3$, 300 K) for compound **7**.

Diethyl 2-(4-(diethylamino)-2-(propionyloxy)benzylidene)malonate, Compound 8:

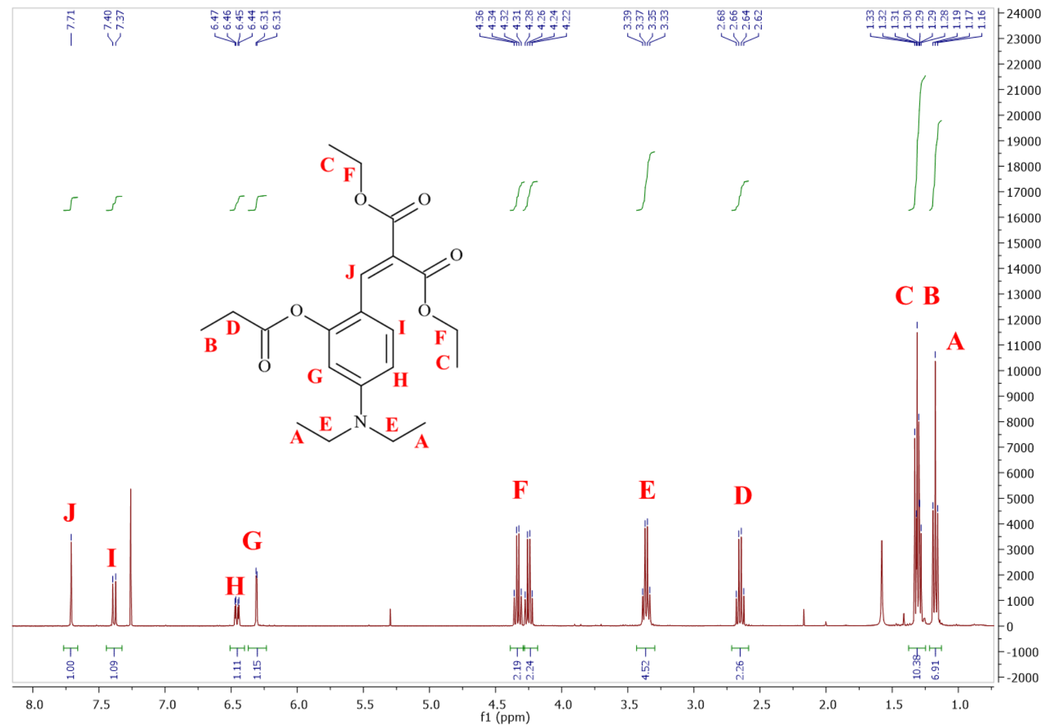

Figure S33: ^{1}H NMR ($CDCl_3$, 300 K) for compound **8**.

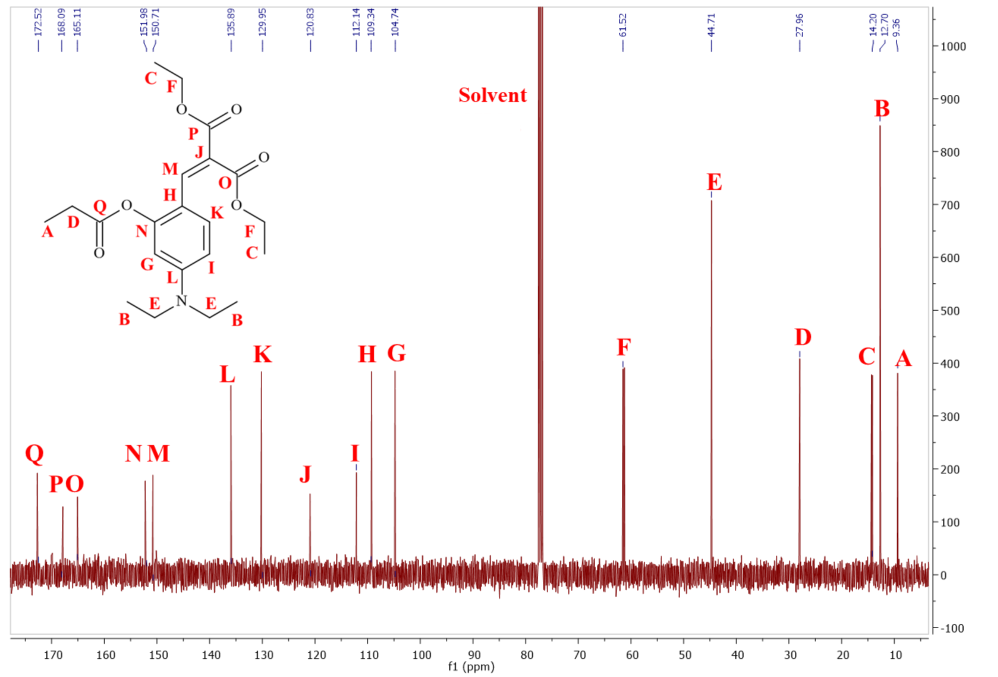

Figure S34: ^{13}C NMR ($CDCl_3$, 300 K) for compound **8**.

Ethyl 7-(diethylamino)-2-oxo-2H-chromene-3-carboxylate, Compound 11:

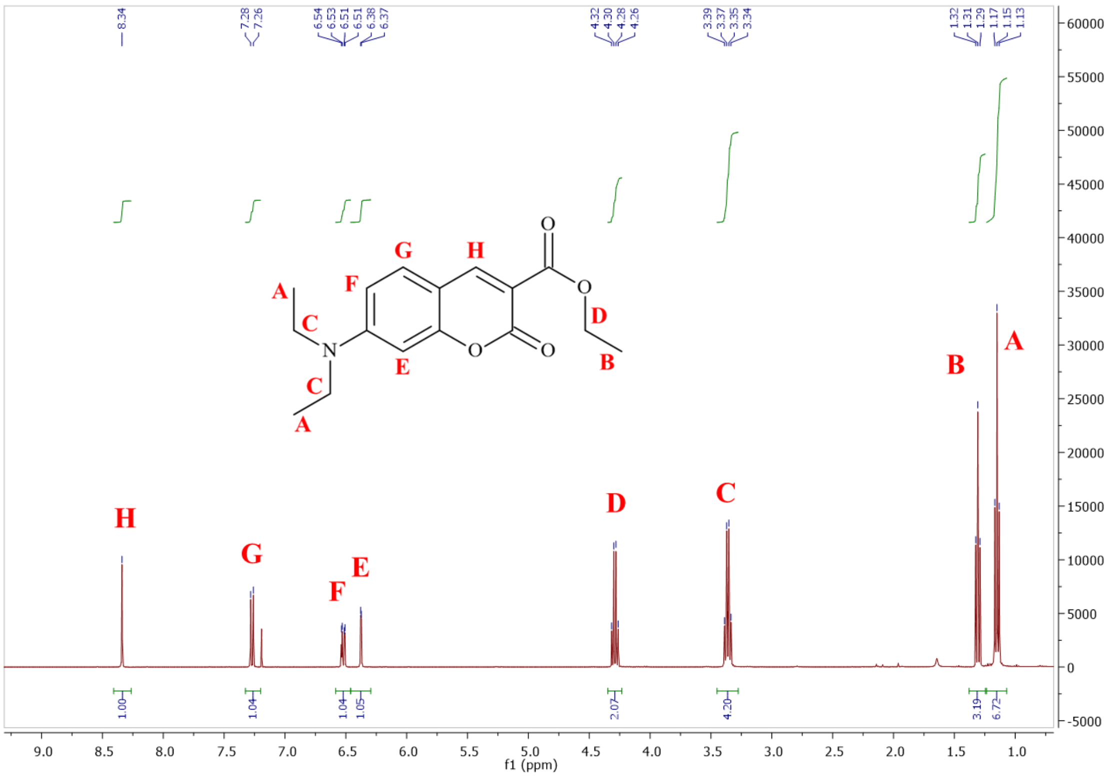

Figure S35: ^{1}H NMR ($CDCl_3$, 300 K) for compound **11**.

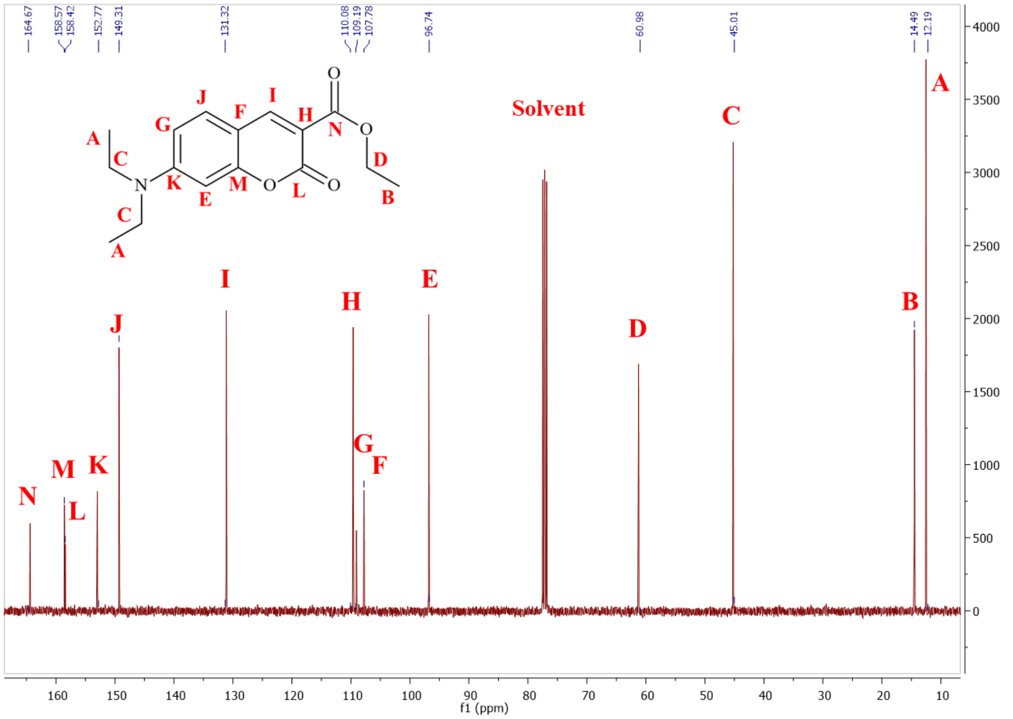

Figure S36: ^{13}C NMR ($CDCl_3$, 300 K) for compound **11**.

Diethyl 2-(4-(diethylamino)-2-((4-oxopentanoyl)oxy)benzylidene)malonate, Compound 9:

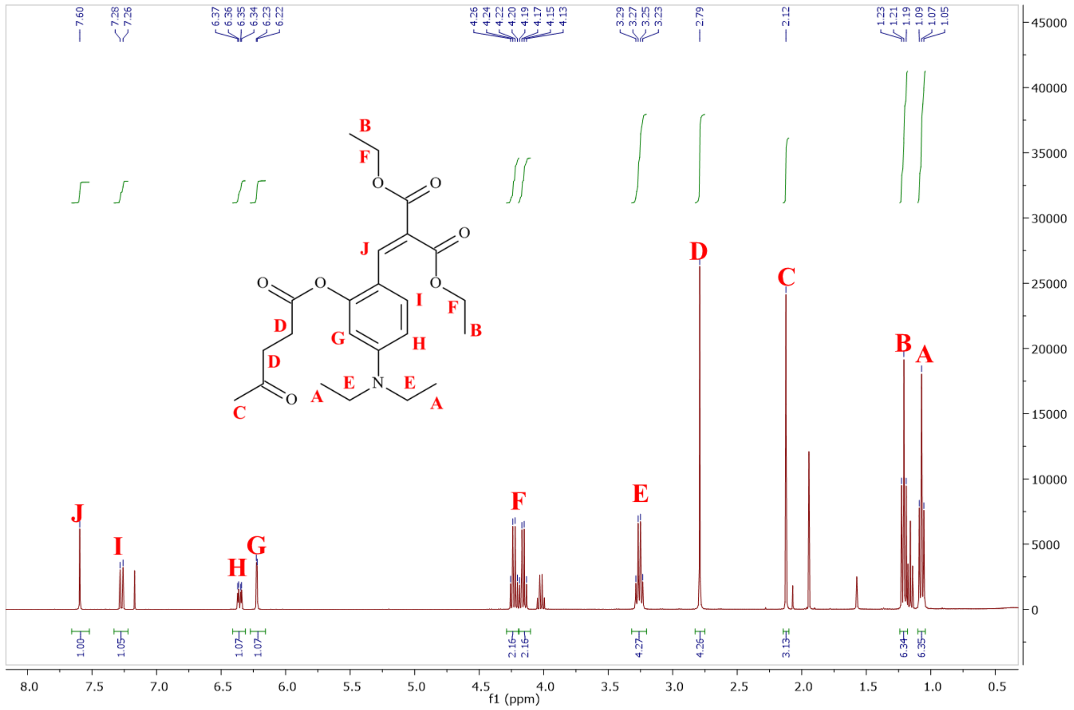

Figure S37: ^{1}H NMR ($CDCl_3$, 300 K) for compound **9**.

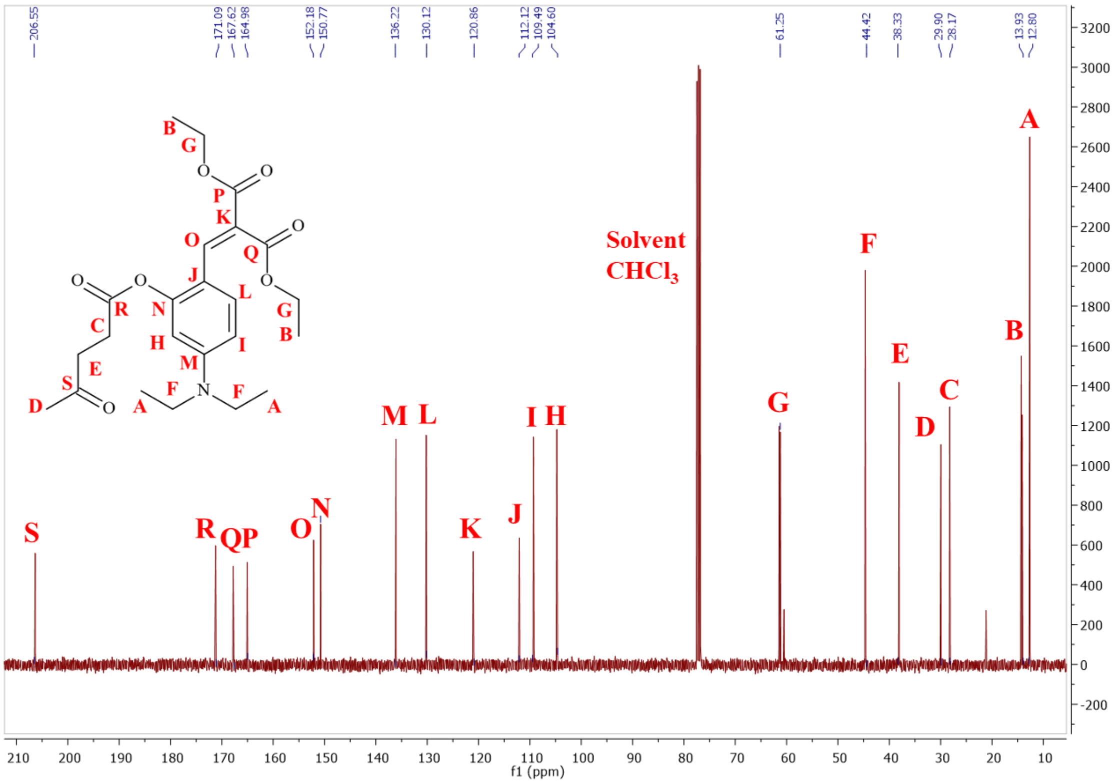

Figure S38: ^{13}C NMR ($CDCl_3$, 300 K) for compound **9**.

Diethyl 2-(2-((4-bromobutanoyl)oxy)-4-(diethylamino)benzylidene)malonate, Compound 10:

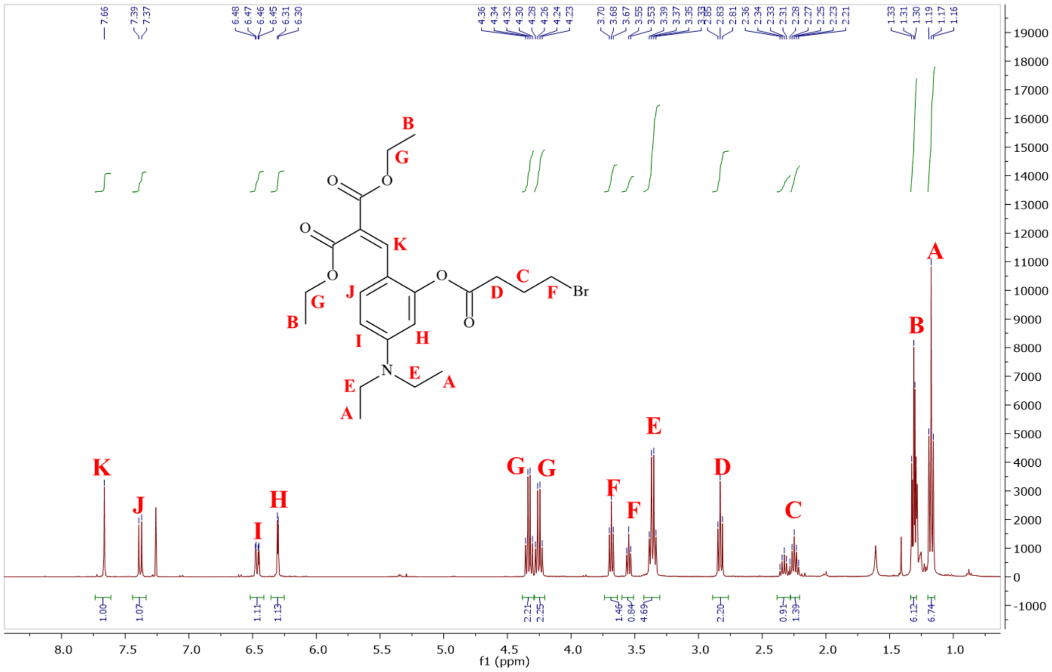

Figure S39: ^{1}H NMR ($CDCl_3$, 300 K) for compound **10**.

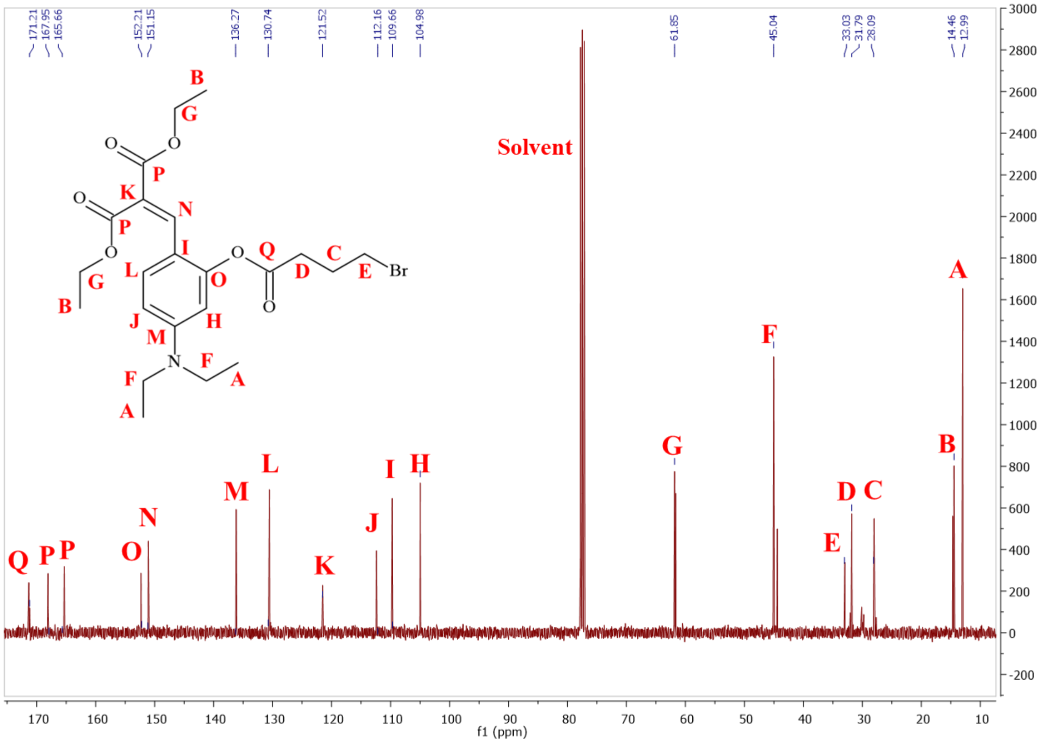

Figure S40: ^{13}C NMR ($CDCl_3$, 300 K) for compound **10**.

Reaction Mechanisms

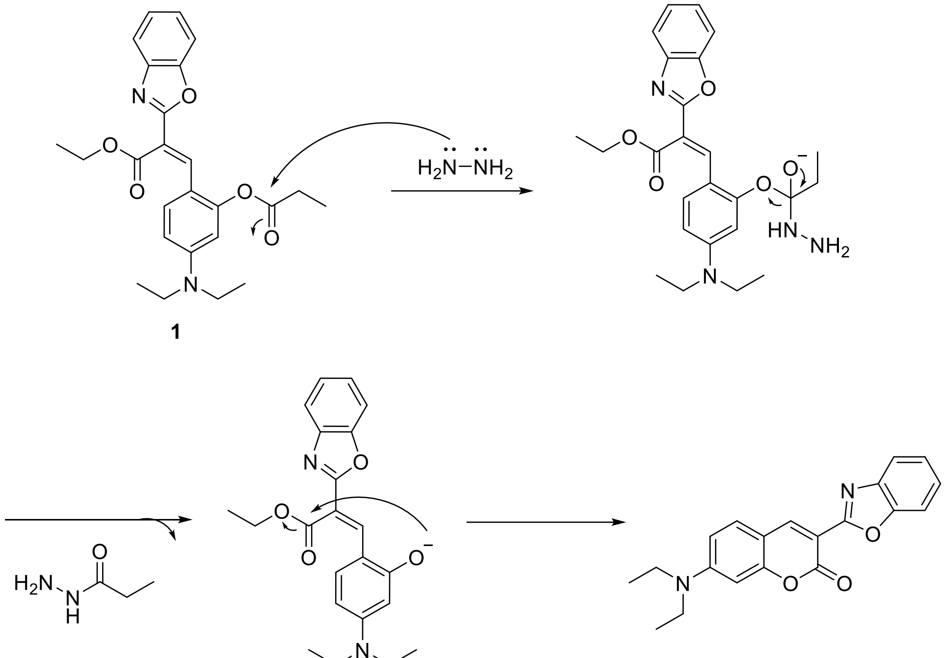

Figure S41: Proposed mechanism path for compound **4** from **1**.

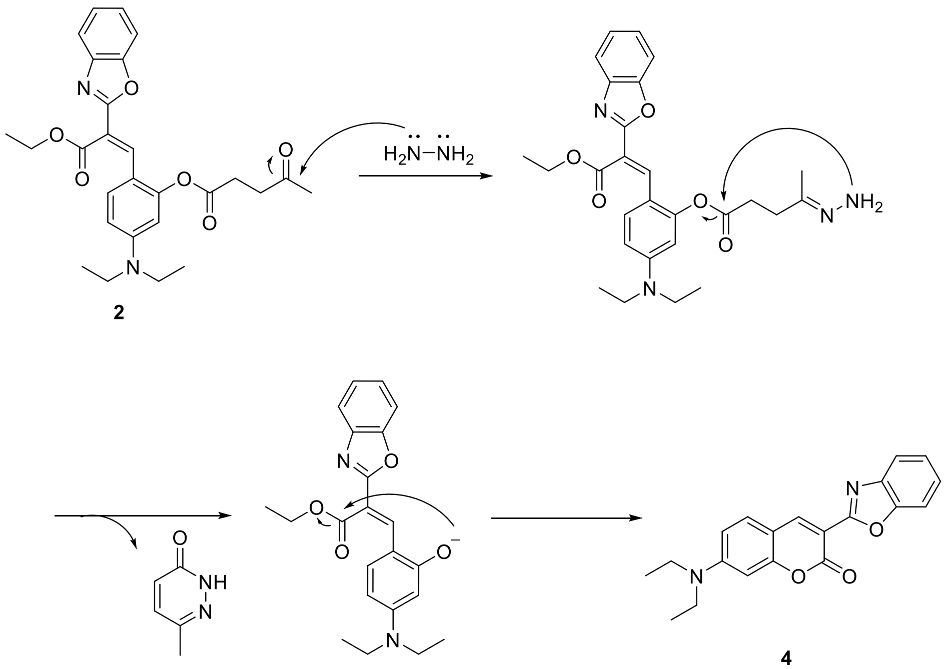

Figure S42: Proposed mechanism for compound **4** from **2**.

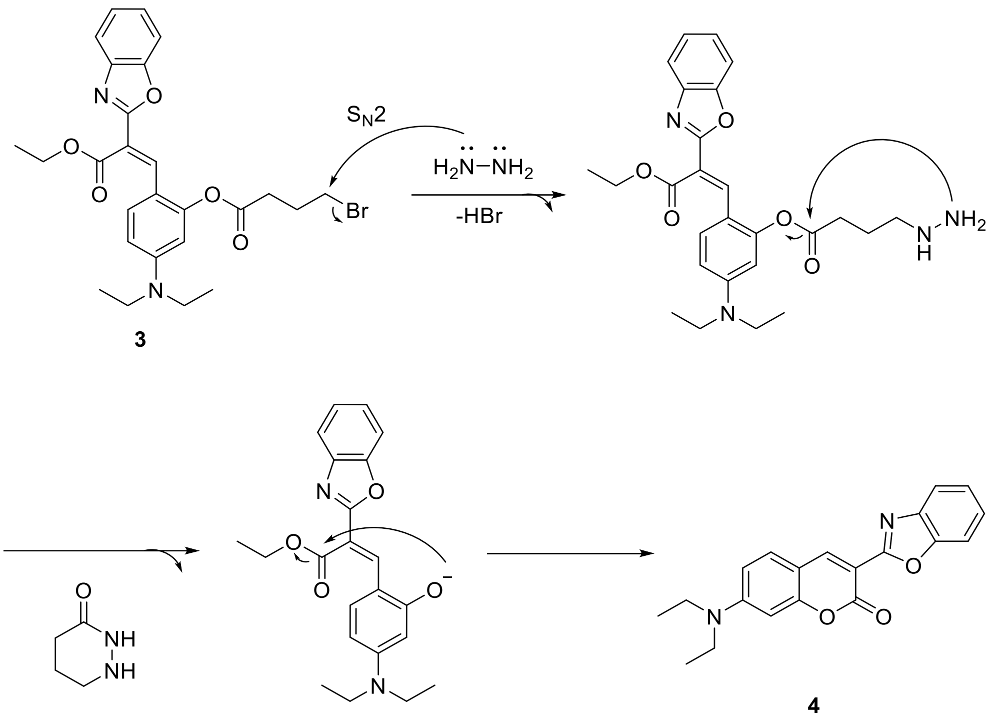

Figure S43: Proposed mechanism for compound **4** from **3**.

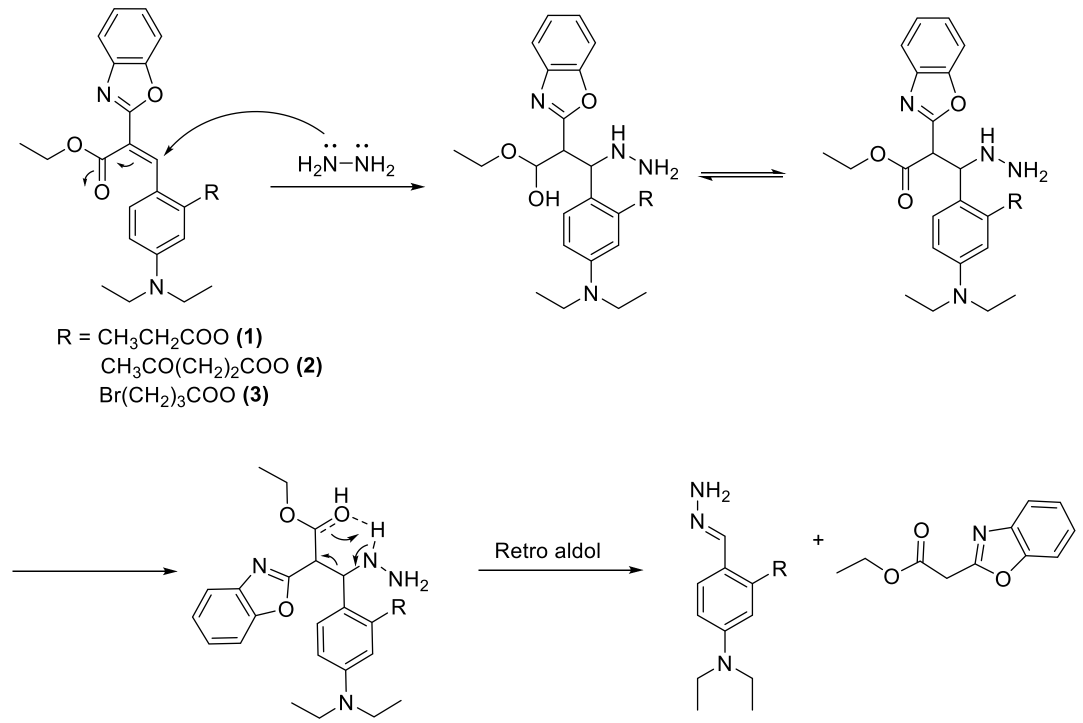

Figure S44: Proposed mechanism for the retro-Knoevenagel reaction.

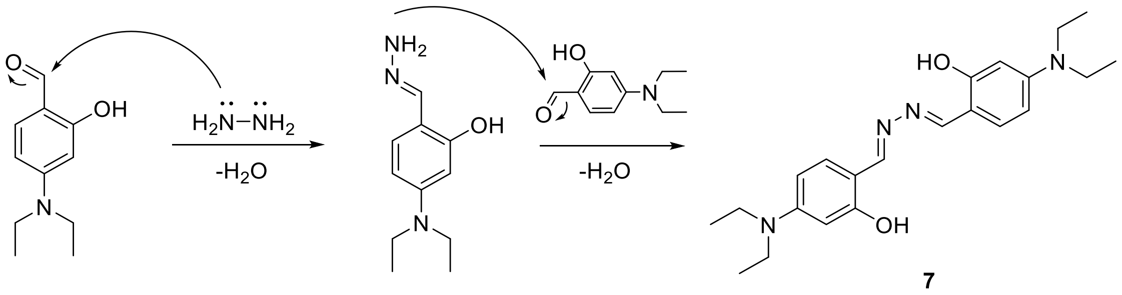

Figure S45: Proposed mechanism path for the formation of **7**.

UV-vis and Fluorescence Results

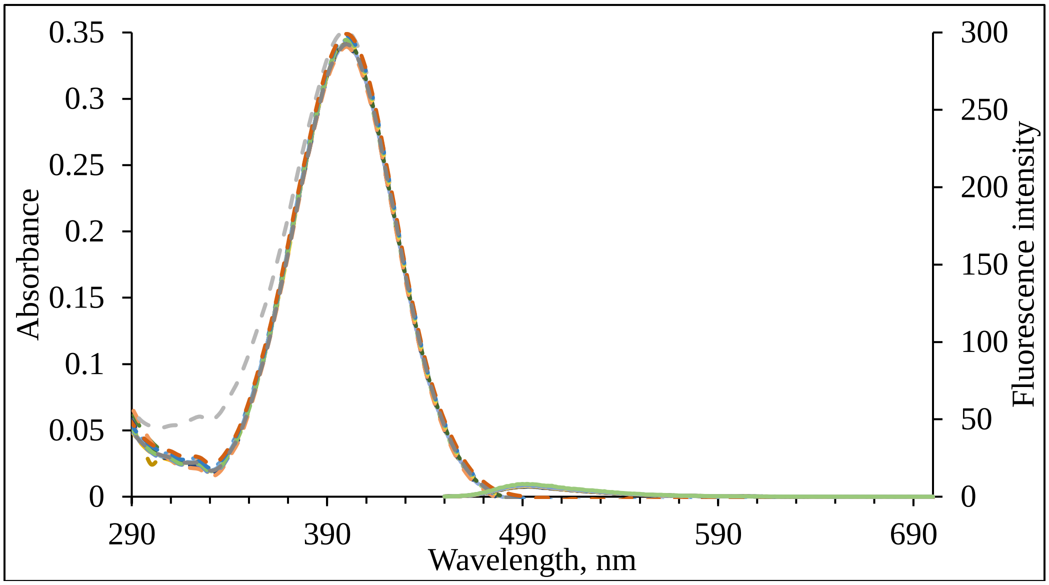

Figure S46: Absorbance (dashed lines) and fluorescence (solid lines) of 10 µM **1** in water-acetonitrile (v/v = 1:9) upon addition of 1.0 equiv different analytes: TBACl, TBABr, TBAI, TBAF, $TBANO_3$, $TBAClO_4$, NaCl, KCl, $MgCl_2$, $NiCl_2$, $CoCl_2$, $CuCl_2$, Alanine, Cysteine, Lysine, ethylamine, 2-aminothiophenol, Thiourea, N_2H_4 (λ_{ex} = 443 nm).

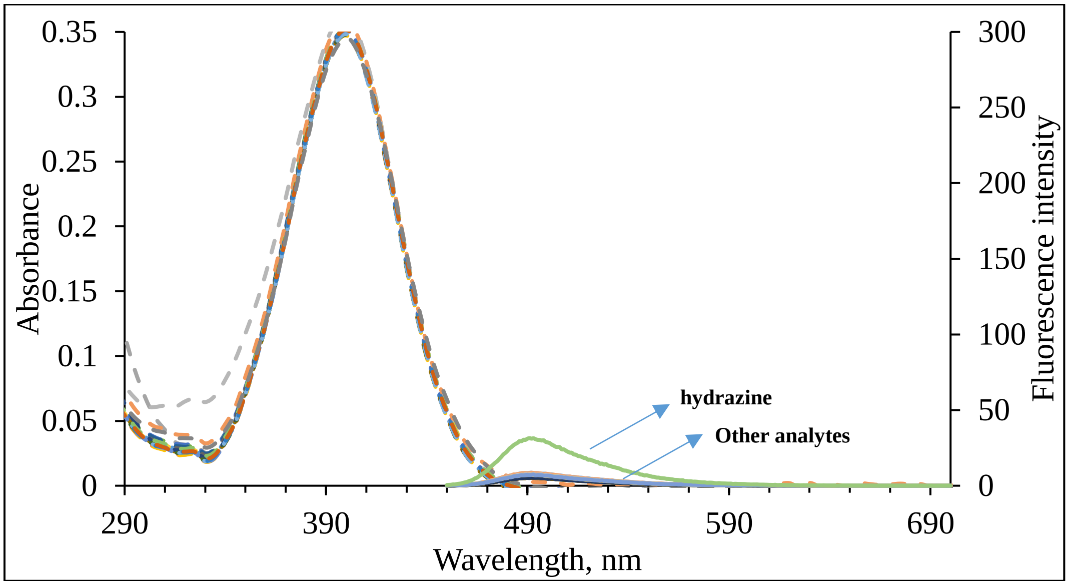

Figure S47: Absorbance (dashed lines) and fluorescence (solid lines) of 10 µM **2** in water-acetonitrile (v/v = 1:9) upon addition of 1.0 equiv different analytes: TBACl, TBABr, TBAI, TBAF, $TBANO_3$, $TBAClO_4$, NaCl, KCl, $MgCl_2$, $NiCl_2$, $CoCl_2$, $CuCl_2$, Alanine, Cysteine, Lysine, ethylamine, 2-aminothiophenol, Thiourea, N_2H_4 (λ_{ex} = 443 nm).

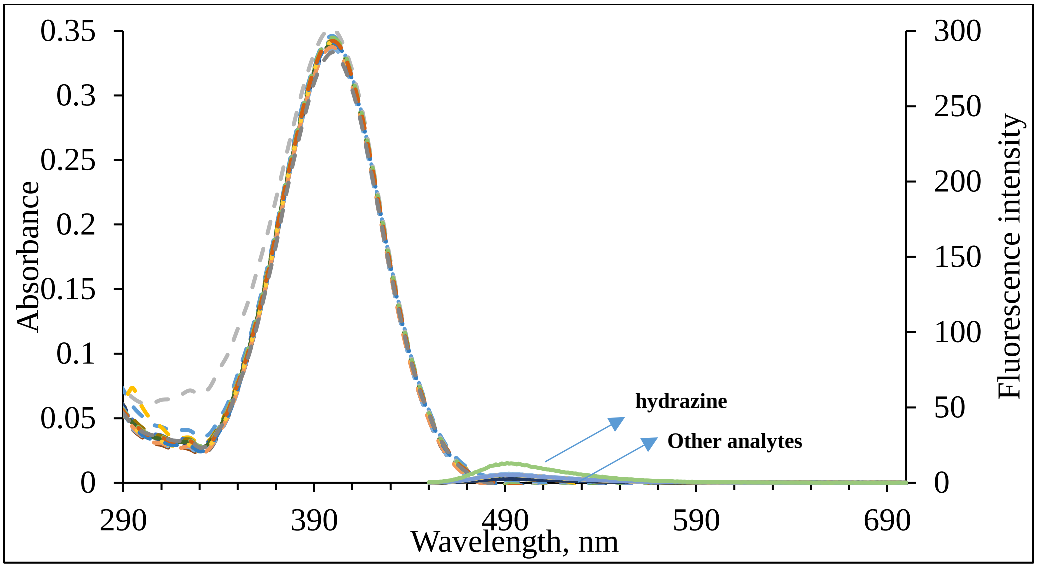

Figure S48: Absorbance (dashed lines) and fluorescence (solid lines) of 10 µM **3** in water-acetonitrile (v/v = 1:9) upon addition of 1.0 equiv different analytes: TBACl, TBABr, TBAI, TBAF, $TBANO_3$, $TBAClO_4$, NaCl, KCl, $MgCl_2$, $NiCl_2$, $CoCl_2$, $CuCl_2$, Alanine, Cysteine, Lysine, ethylamine, 2-aminothiophenol, Thiourea, N_2H_4 (λ_{ex} = 443 nm).

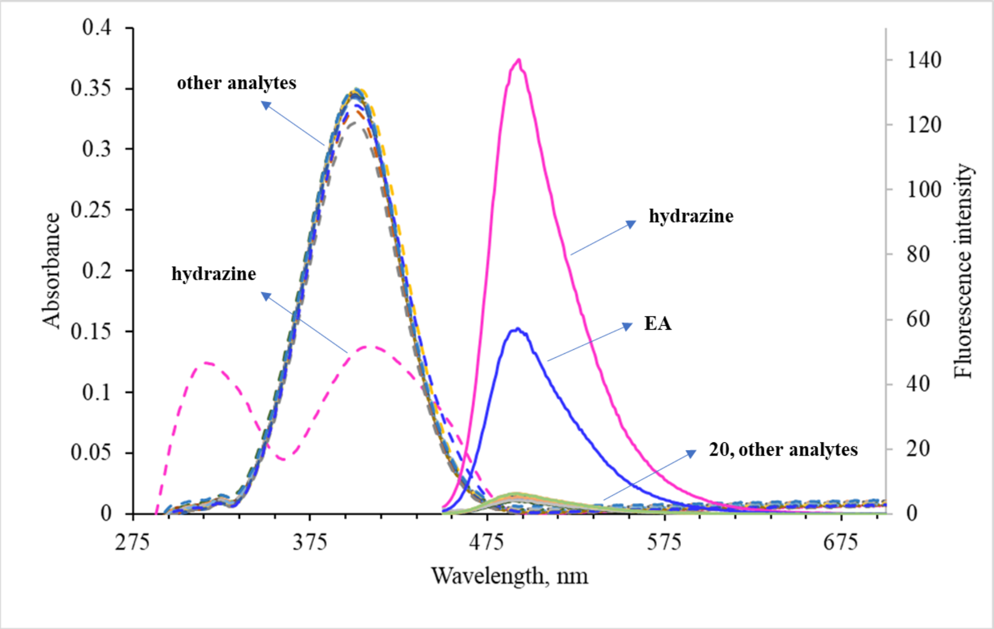

Figure S49: Absorbance (dashed lines) and fluorescence (solid lines) of 10 µM **1** in water-acetonitrile (v/v = 1:9) upon addition of 30.0 equiv different analytes: TBACl, TBABr, TBAI, TBAF, $TBANO_3$, $TBAClO_4$, NaCl, KCl, $MgCl_2$, $NiCl_2$, $CoCl_2$, $CuCl_2$, Alanine, Cysteine, Lysine, ethylamine, 2-aminothiophenol, Thiourea, N_2H_4 (λ_{ex} = 443 nm).

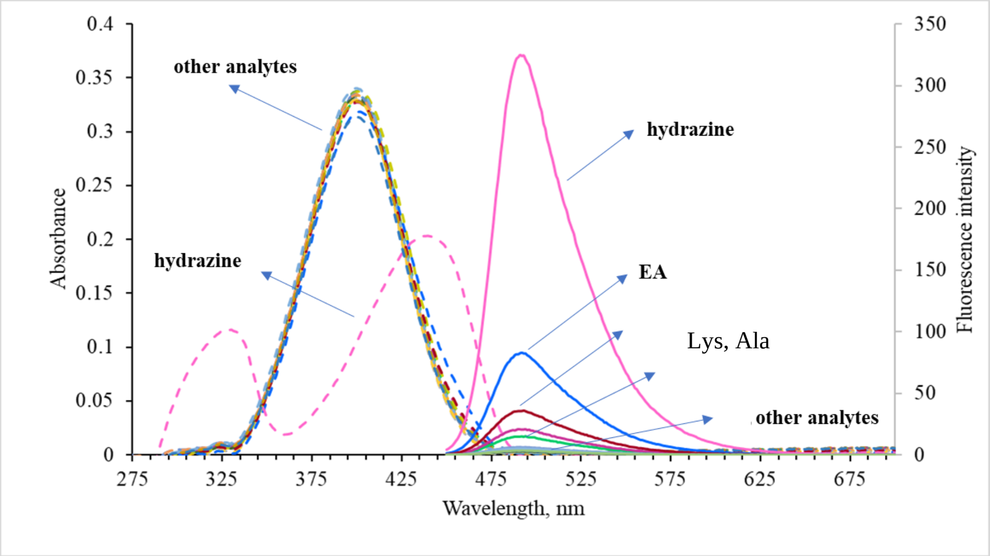

Figure S50: Absorbance (dashed lines) and fluorescence (solid lines) of 10 μM **2** in water-acetonitrile (v/v = 1:9) upon addition of 30.0 equiv different analytes: TBACl, TBABr, TBAI, TBAF, $TBANO_3$, $TBAClO_4$, NaCl, KCl, $MgCl_2$, $NiCl_2$, $CoCl_2$, $CuCl_2$, Alanine, Cysteine, Lysine, ethylamine, 2-aminothiophenol, Thiourea, N_2H_4 (λ_{ex} = 443 nm).

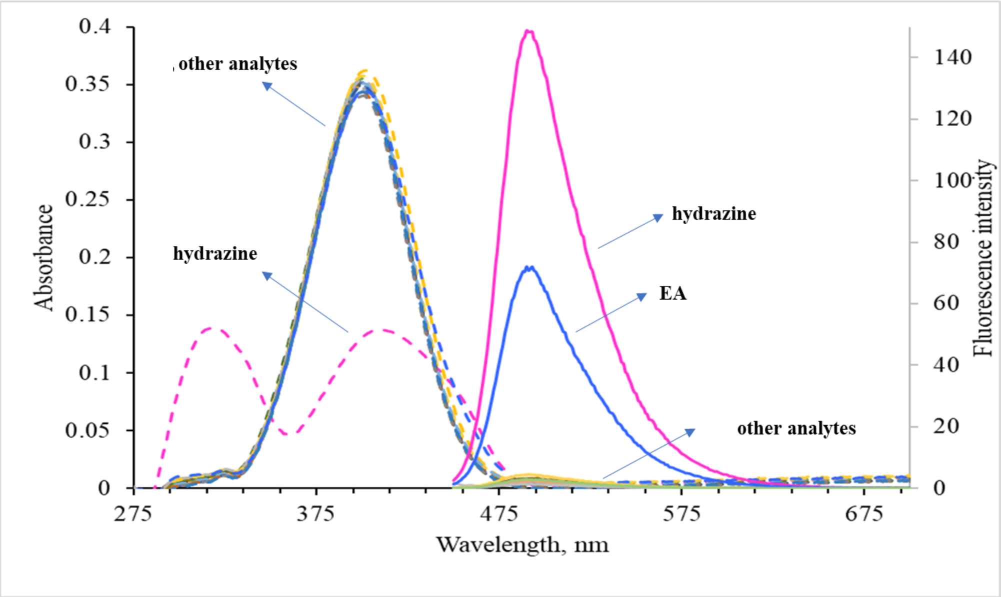

Figure S51: Absorbance (dashed lines) and fluorescence (solid lines) of 10 μM **3** in water-acetonitrile (v/v = 1:9) upon addition of 30.0 equiv different analytes: TBACl, TBABr, TBAI, TBAF, $TBANO_3$, $TBAClO_4$, NaCl, KCl, $MgCl_2$, $NiCl_2$, $CoCl_2$, $CuCl_2$, Alanine, Cysteine, Lysine, ethylaminme, 2-aminothiophenol, Thiourea, N_2H_4 (λ_{ex} = 443 nm).

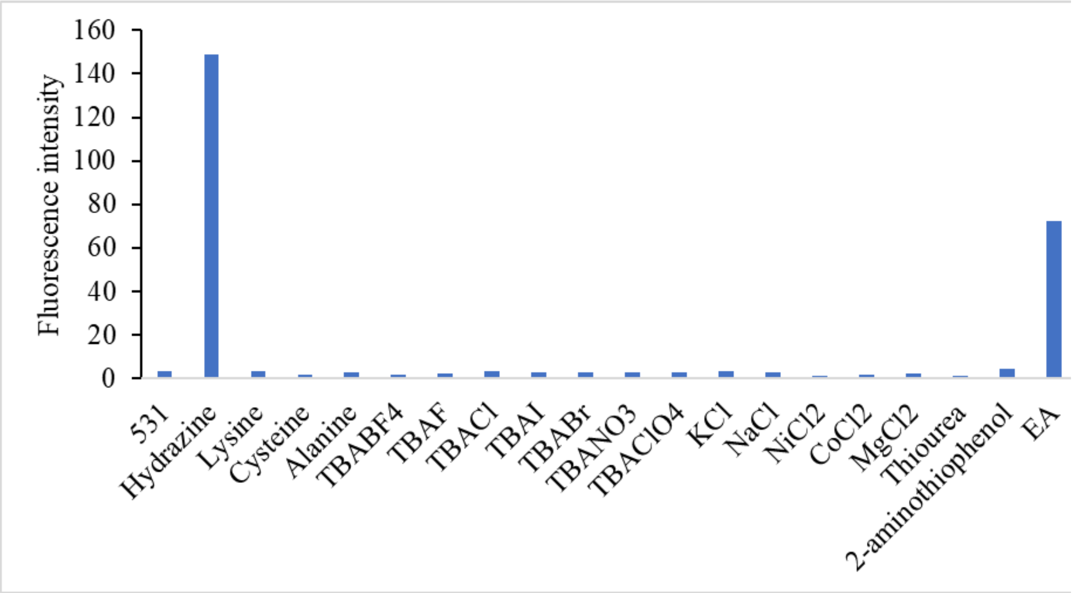

Figure S52. Bar graph of fluorescence intensity for 10 µM **3** in water-acetonitrile (v/v = 1:9) upon addition of 30.0 equiv different analytes: TBACl, TBABr, TBAI, TBAF, $TBANO_3$, $TBAClO_4$, NaCl, KCl, $MgCl_2$, $NiCl_2$, $CoCl_2$, $CuCl_2$, Alanine, Cysteine, Lysine, ethylamine, 2-aminothiophenol, Thiourea, N_2H_4 (λ_{ex} = 443 nm).

LOD studies

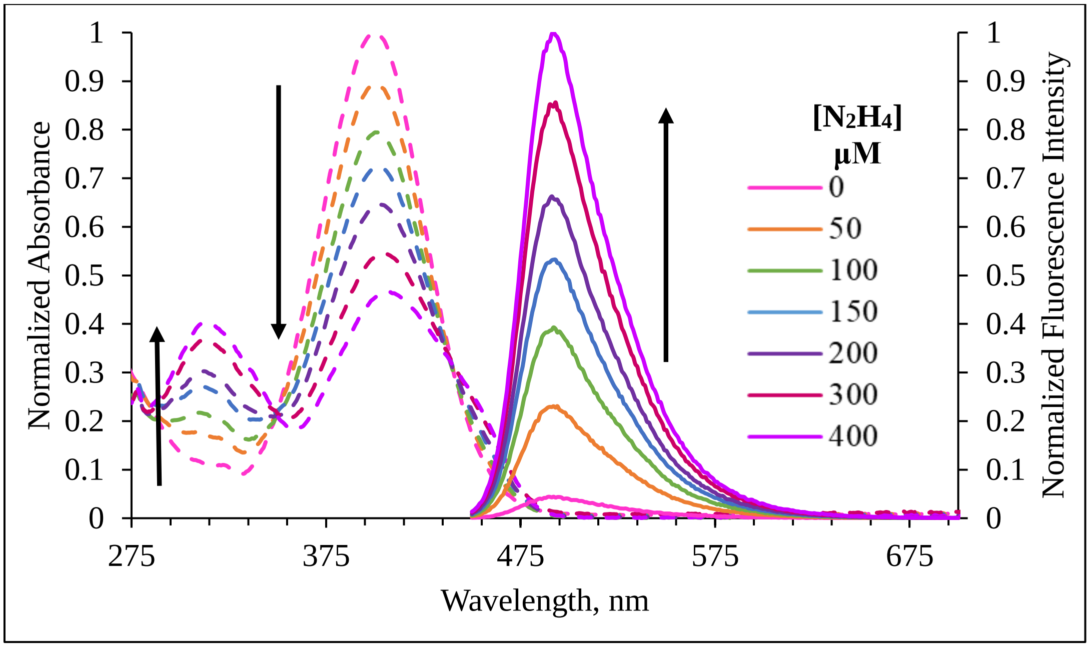

Figure S53: Absorbance (dashed lines) and fluorescence (solid lines) of 10 µM **1** in water-acetonitrile (v/v = 1:9) upon addition of 0-30.0 equiv N_2H_4 (λ_{ex} = 443 nm).

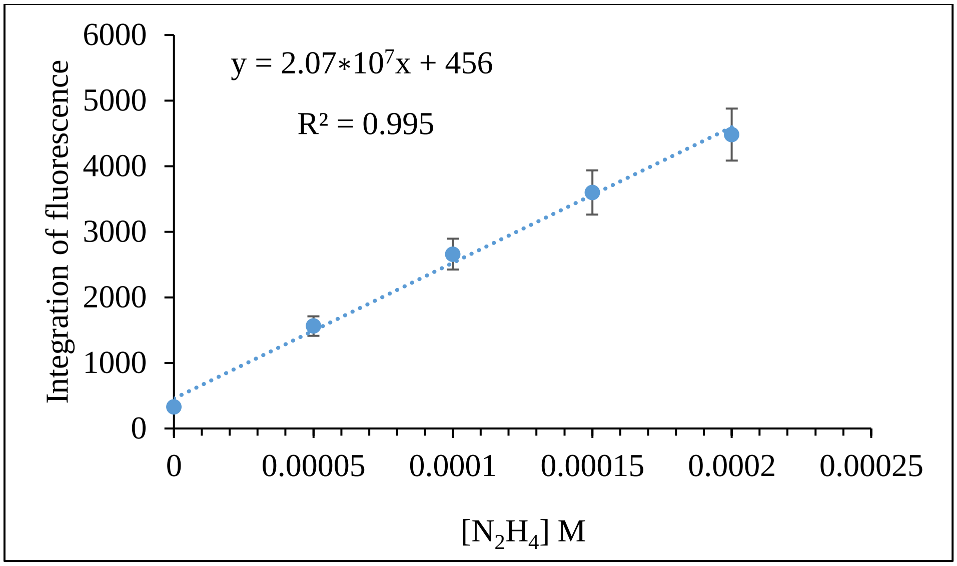

Figure S54: Fluorescence integration of 10 μM **1** in water-acetonitrile (v/v = 1:9) as function of N_2H_4 concentration (λ_{ex} = 443 nm).

The linear equation was found to be y = 2.07*10^7x + 456 (R^2 = 0.995) and the detection limit was calculated, LOD = 2.7 (2) μM.

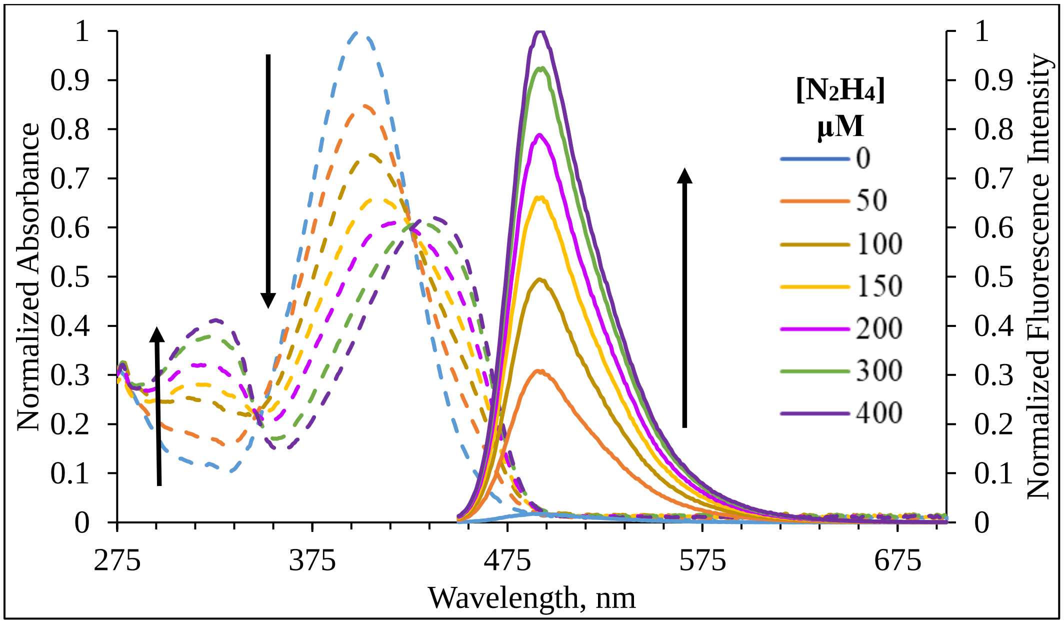

Figure S55: Absorbance (dashed lines) and fluorescence (solid lines) of 10 μM **2** in water-acetonitrile (v/v = 1:9) upon addition of 0-30.0 equiv N_2H_4 (λ_{ex} = 443 nm).

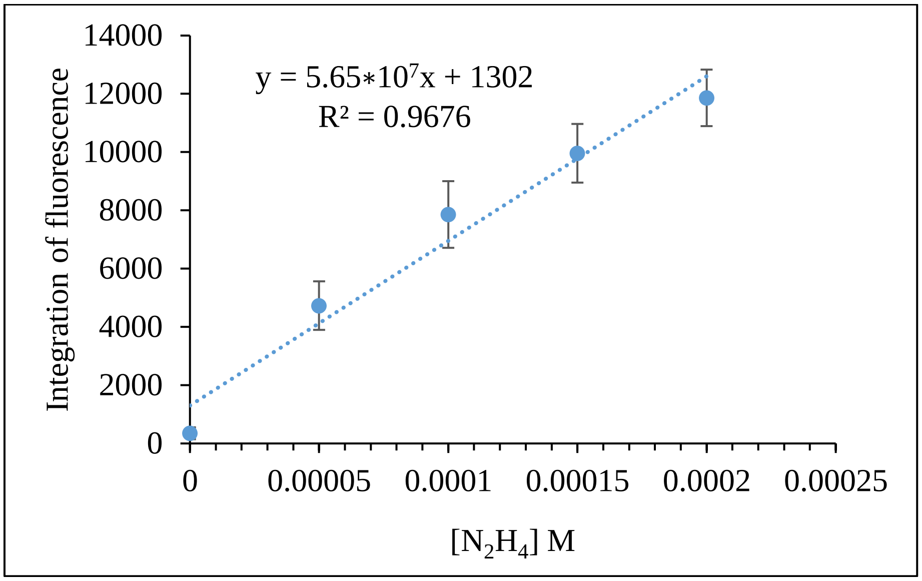

Figure S56: Fluorescence integration of 10 µM **2** in water-acetonitrile (v/v = 1:9) as function of N_2H_4 concentration (λ_{ex} = 443 nm).

The linear equation was found to be y = 5.65∗ 10^7x + 1302 (R^2 = 0.9676) and the limit of detection was calculated, LOD = 0.84 (1) µM.

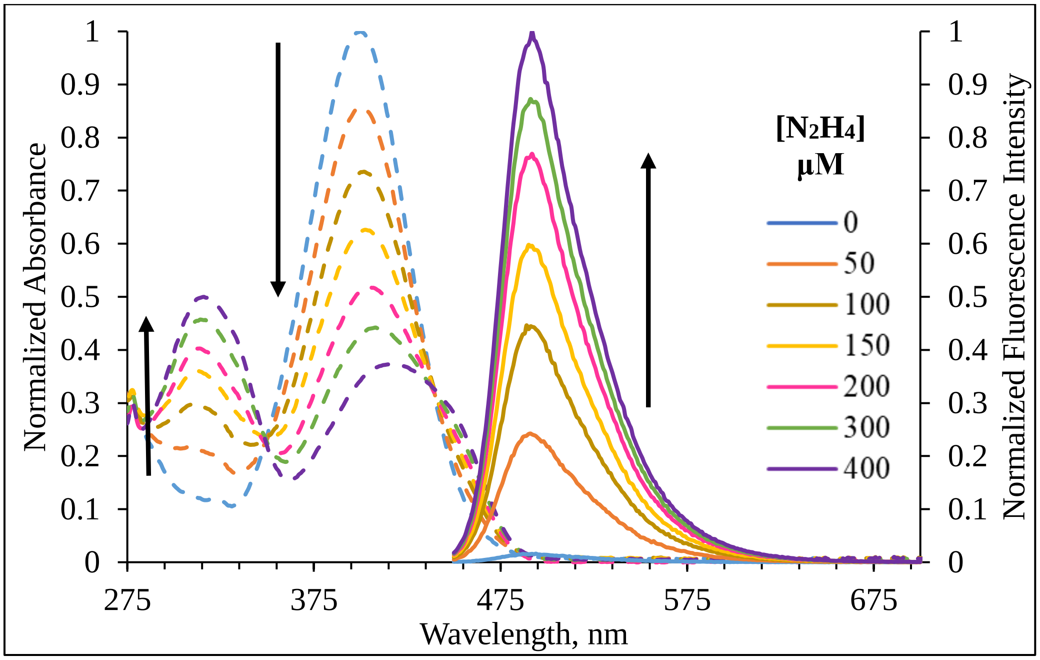

Figure S57: Absorbance (dashed lines) and fluorescence (solid lines) of 10 µM **3** in water-acetonitrile (v/v = 1:9) upon addition of 0-30.0 equiv N_2H_4 (λ_{ex} = 443 nm).

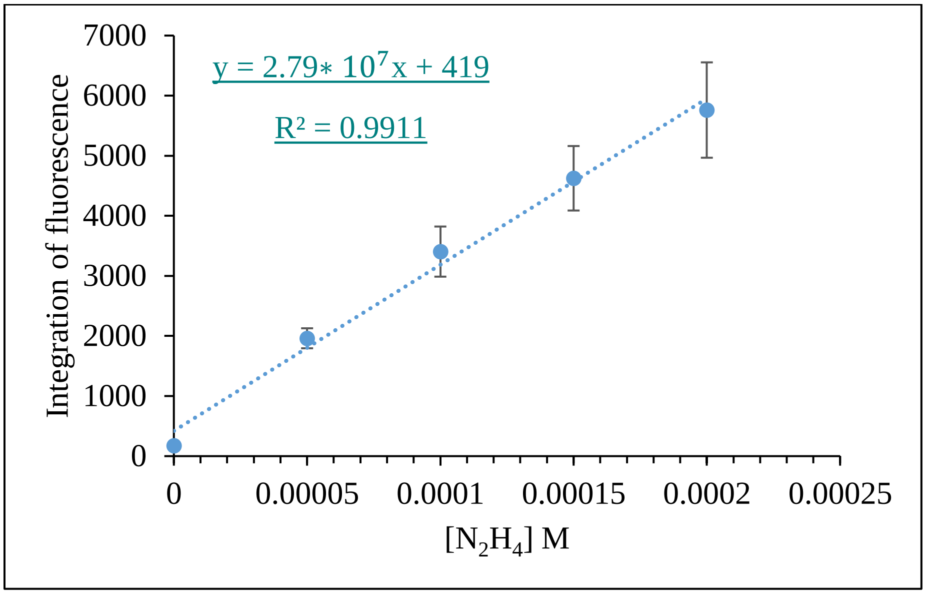

Figure S58: Fluorescence integration of 10 µM **3** in water-acetonitrile (v/v = 1:9) as function of N_2H_4 concentration (λ_{ex} = 443 nm).

The linear equation was identified as $y = 2.79 * 10^7x + 419$ ($R^2 = 0.9911$) and the LOD was determined to be 1.8 (3) µM.

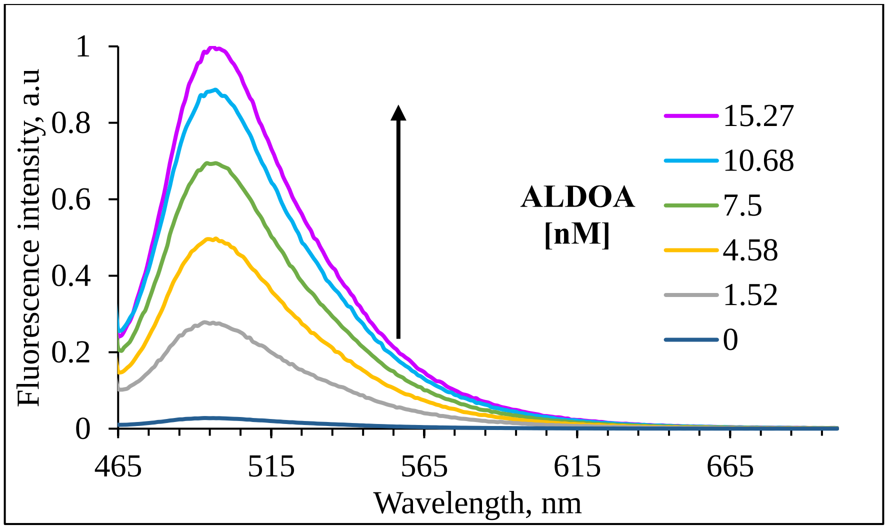

Figure 159: Normalized fluorescence of 5.0 µM **3** in 1% MeCN – 99% PBS buffer pH 7.4 upon addition of 0- 15.27 nM ALDOA (λ_{ex} = 455 nm).

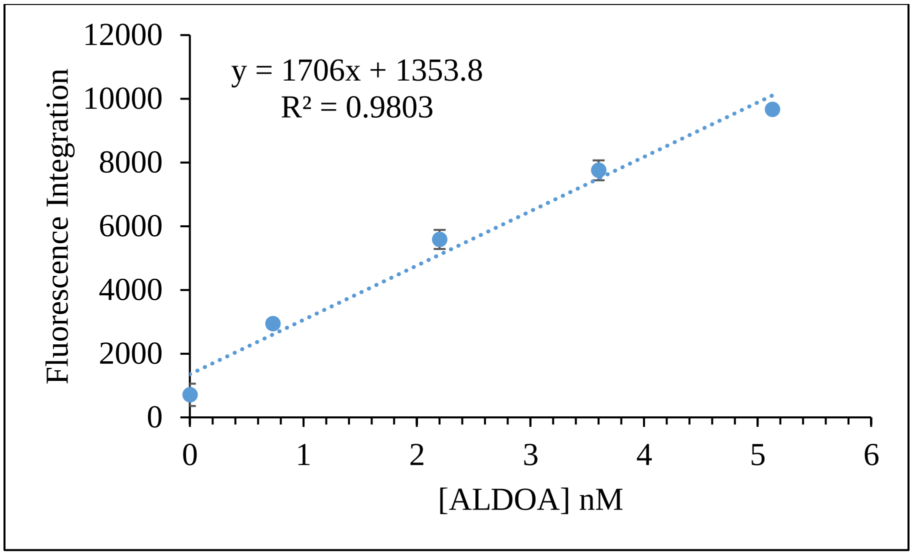

Figure S60: Fluorescence integration of 5 µM **3** in in 1% MeCN – 99% PBS buffer pH 7.4 as function of ALDOA concentration 0- 15.27 nM (λ_{ex} = 455 nm).

The linear equation was found to be y = 1706x + 1353.8 (R^2 = 0.9803) and the limit of detection was calculated to be 0.063 (6) nM.

*Comparison of a real sample of **4** to the product of the reaction between **2** and hydrazine, an NMR study.*

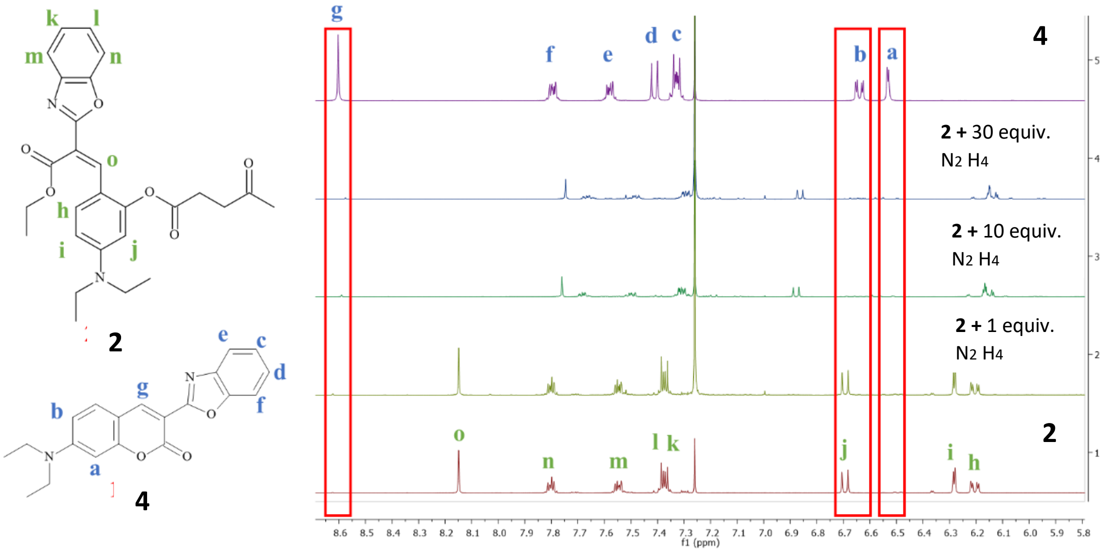

Figure S61: Stacked and zoomed in, ^{1}H NMR spectra of **4**, and **2** upon addition of 0, 1.0, 15.0 and 30.0 equiv. N_2H_4.
